# Comparative efficacy of tirzepatide and glucagon-like peptide-1 receptor agonists on cardiovascular outcomes in patients with type 2 diabetes: a systematic review and network meta-analysis

**DOI:** 10.1186/s12933-026-03113-3

**Published:** 2026-02-27

**Authors:** Arveen Shokravi, Jayant Seth, G.B. John Mancini

**Affiliations:** 1 Department of Medicine, Faculty of Medicine, The University of British Columbia, 6200 University Blvd, Vancouver, BC V6T1Z3 Canada; 2 Department of Medicine, Cumming School of Medicine, Health Sciences Centre, University of Calgary, 3330 Hospital Drive NW, Calgary, AB T2N4N1 Canada; 3 Centre for Cardiovascular Innovation, Dilwari Cardiovascular Institute, Division of Cardiology, University of British Columbia, Vancouver, BC V5Z 1M9 Canada

**Keywords:** tirzepatide, glucagon-like peptide-1 receptor agonists, type 2 diabetes, cardiovascular outcomes, meta-analysis

## Abstract

**Background:**

Glucagon-like peptide-1 receptor agonists (GLP-1RAs) are widely used therapies for cardiovascular risk reduction in type 2 diabetes (T2D). With the emergence of the SURPASS-CVOT trial, tirzepatide (a dual GIP/GLP-1 receptor agonist) has entered the therapeutic landscape; however, its comparative effect on cardiovascular outcomes compared to placebo and individual GLP-1RAs remains undefined.

**Methods:**

We conducted a systematic review and frequentist network meta-analysis (NMA) of RCTs enrolling adults with type 2 diabetes (T2D) and established atherosclerotic cardiovascular disease (ASCVD) or high cardiovascular (CV) risk. Eligible RCTs evaluated tirzepatide or GLP-1RAs and reported major adverse cardiovascular events (MACE), CV mortality, all-cause mortality, non-fatal myocardial infarction (MI) or non-fatal stroke. A class-level NMA was conducted to estimate the incremental benefit of tirzepatide and GLP-1RAs over placebo, and an agent-level NMA was conducted to explore differences between tirzepatide and individual GLP-1RA agents. Subgroup analyses, including established cardiovascular disease populations, and leave-one-out sensitivity analyses were performed.

**Results:**

Eleven trials met inclusion criteria (10 GLP-1RA trials and 1 tirzepatide trial [SURPASS-CVOT]). In the class-level analysis, tirzepatide significantly reduced MACE (HR 0.79, 95% CI 0.69–0.91), CV mortality (HR 0.77, 95% CI 0.66–0.90), all-cause mortality (HR 0.74, 95% CI 0.65–0.83), non-fatal MI (HR 0.77, 95% CI 0.61–0.97), and non-fatal stroke (HR 0.79, 95% CI 0.64–0.97) compared to placebo. Formal statistical comparisons between tirzepatide and the GLP-1RA class could not be performed within the constraints of the NMA; however, point estimates across outcomes numerically favored tirzepatide compared with placebo. In the agent-level analysis, tirzepatide reduced MACE compared to placebo (HR 0.81, 95% CI 0.70–0.94) and lixisenatide (HR 0.79, 95% CI 0.65–0.97). Subgroup and sensitivity analyses did not substantially change point estimates.

**Conclusion:**

Among adults with T2D and established ASCVD or high CV risk, class-level analysis demonstrated that tirzepatide significantly reduced the risk of cardiovascular events compared to placebo; at the agent-level, tirzepatide demonstrated comparable efficacy to individual GLP-1RAs. These findings suggest that tirzepatide provides cardiovascular benefit at least comparable to established GLP-1RAs, supporting its emerging role in cardiovascular risk reduction in T2D.

**Graphical abstract:**

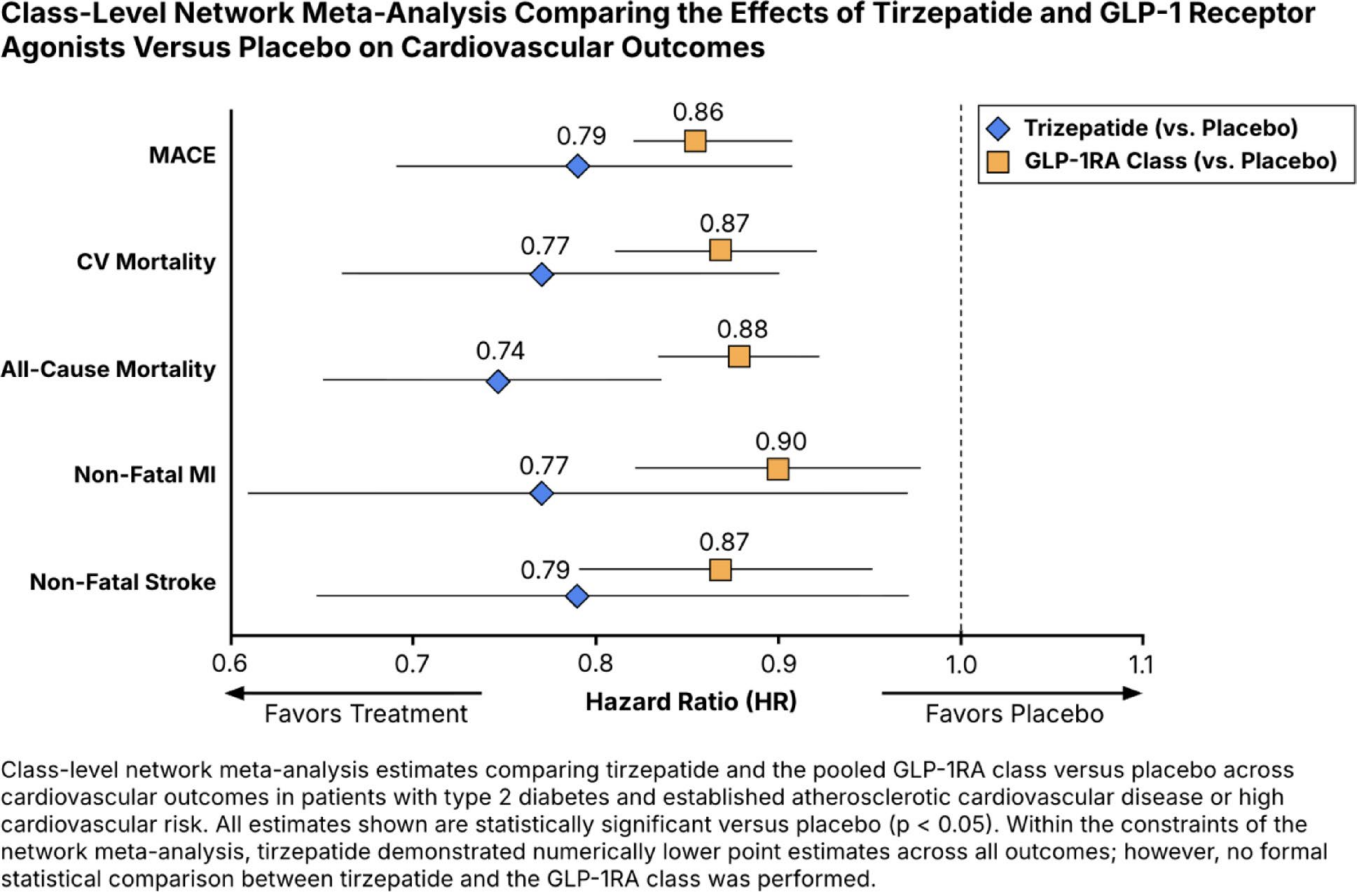

**Supplementary Information:**

The online version contains supplementary material available at 10.1186/s12933-026-03113-3.

## Introduction

Over the past decade, glucagon-like peptide-1 receptor agonists (GLP-1RAs) have demonstrated cardiovascular, renal, and metabolic benefits in patients with type 2 diabetes (T2D) and atherosclerotic cardiovascular disease (ASCVD) or high cardiovascular (CV) risk, and have subsequently been incorporated into multiple international guidelines [1–3].

Given the frequent coexistence of obesity and T2D with cardiovascular disease and chronic kidney disease (CKD), incretin-based therapies (encompassing selective GLP-1 and multi-receptor agonists) have emerged as disease-modifying treatments across the cardiovascular–kidney–metabolic (CKM) spectrum. Tirzepatide has emerged as a novel incretin-based therapy, functioning both as a glucose-dependent insulinotropic polypeptide (GIP) and GLP-1RA [4]. The SURPASS trials established tirzepatide’s glycemic, cardiometabolic, and renal benefits in T2D, while the SURMOUNT trials demonstrated tirzepatide’s efficacy in weight reduction in populations with obesity [4–6]. A pre-specified meta-analysis of seven SURPASS trials demonstrated that tirzepatide was not inferior to placebo or active comparators, including insulin glargine, insulin degludec, semaglutide, and dulaglutide, in major adverse cardiovascular event (MACE) reduction [7].

Tirzepatide’s dual mechanism has prompted investigation into whether it confers incremental cardiovascular benefits beyond those observed with GLP-1RAs. The SURPASS-CVOT trial assessed tirzepatide’s effects on cardiovascular endpoints in patients with T2D and established ASCVD, incorporating a unique design, using dulaglutide as the active comparator [8]. While SURPASS-CVOT showed that tirzepatide was noninferior to dulaglutide, uncertainty remains regarding how tirzepatide compares to the broader GLP-1RA class and other GLP-1RA agents, and the extent to which it provides incremental benefit over placebo.

To address this, we conducted a systematic review and network meta-analysis (NMA) examining RCTs of patients with T2D and ASCVD or high CV risk, with two primary objectives: (1) To perform a class-level NMA to evaluate the effect of tirzepatide compared with placebo on cardiovascular outcomes; and (2) to perform an agent-level NMA comparing tirzepatide with individual GLP-1RAs, including albiglutide, dulaglutide, efpeglenatide, exenatide, liraglutide, lixisenatide, and semaglutide.

## Methods

This systematic review was conducted in accordance with the PRISMA Extension Statement for Reporting of Systematic Reviews Incorporating Network Meta-analyses of Health Care Interventions [9] guidelines and was prospectively submitted for registration on PROSPERO (registration code: CRD420251157060).

### Search strategy and study selection

A comprehensive search was conducted across MEDLINE and Embase from database inception to October 2025, using the key terms including tirzepatide OR GLP-1 receptor agonist AND type 2 diabetes AND Randomized Controlled Trial. The full search strategy is provided in the Supplementary Appendix (Table S1 and S2).

Two investigators independently screened titles and abstracts using Covidence [10]. Inclusion criteria included RCTs that evaluated GLP-1RAs or tirzepatide in adults with T2D and established ASCVD or high CV risk (including CKD), and reported the following outcomes: MACE, CV mortality, all-cause mortality, non-fatal myocardial infarction (MI), or non-fatal stroke. Exclusion criteria included non-randomized studies, trials enrolling pediatric populations, and studies of less than 1,000 patients or less than 12 month follow up. The 1,000 participants and 12 months follow-up requirements were set as an arbitrary cutoff to ensure adequate event accrual and statistical power. Investigational or device-based incretin delivery systems (e.g., continuous subcutaneous exenatide infusion [ITCA 650]) were excluded to maintain clinical comparability with conventionally administered GLP-1RA formulations used in routine practice and represented in other major CV outcome trials. Discrepancies identified during the screening, full-text, and data extraction process were resolved through consensus.

### Outcomes of interest

**Fig. 1:**
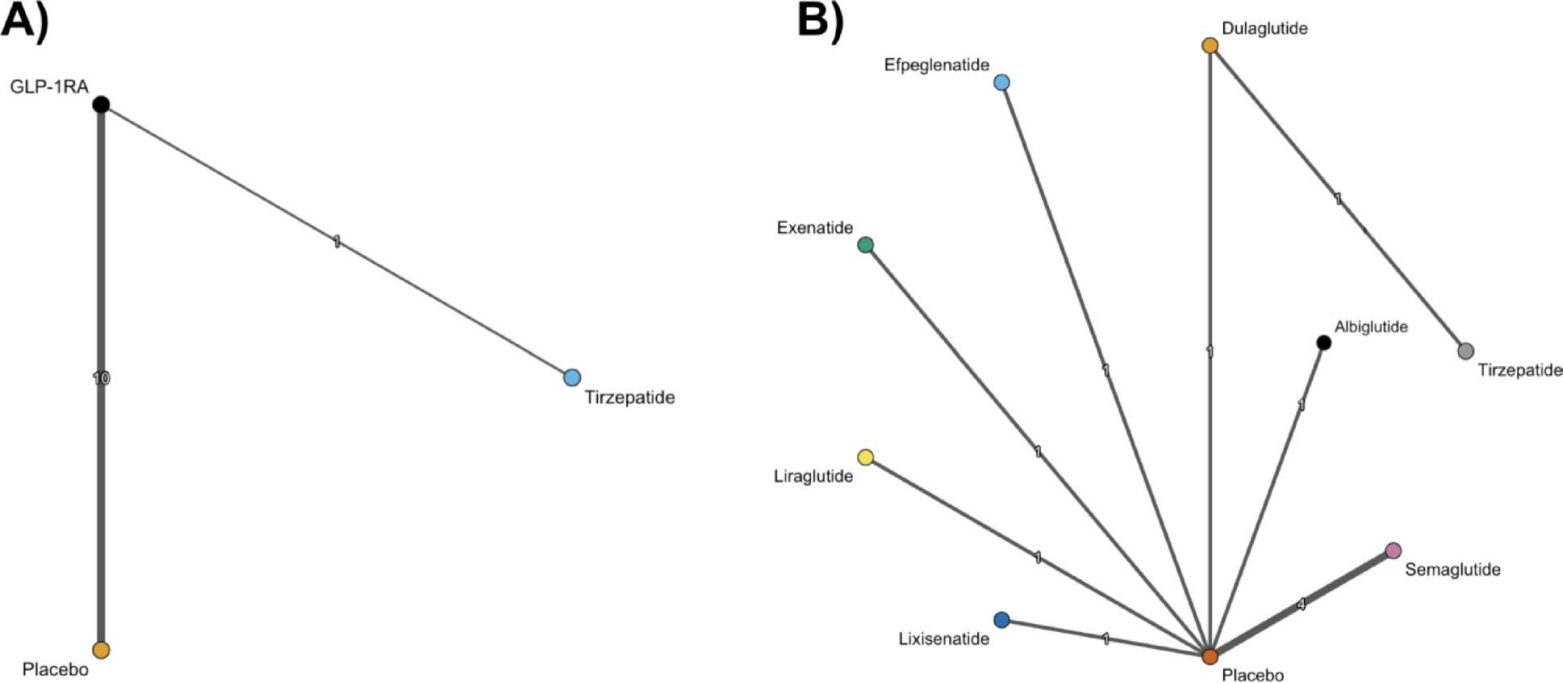
Network structure for network meta-analyses in this study. **A** Class-level NMA comparing tirzepatide, GLP-1RAs as a class, and placebo. **B** Agent-level NMA comparing tirzepatide, individual GLP-1RAs, and placebo

In this study, a hierarchical NMA framework was used, incorporating both class-level and agent-level analyses. The primary analysis was a class-level NMA comparing tirzepatide with placebo, using GLP-1RAs as a pooled class comparator (Fig. 1, Panel A). A secondary agent-level NMA compared tirzepatide with individual GLP-1RAs and placebo (Fig. 1, Panel B). Both analyses were performed across all outcomes of interest.

### Statistical methods

A frequentist NMA was performed using the netmeta package in R [11]. A random-effects model with restricted maximum likelihood (REML) estimation was used to account for between-study heterogeneity, and the Hartung-Knapp adjustment was applied to 95% confidence intervals (CIs). Results are reported as hazard ratios (HRs) with 95% CIs. Between-study variance was quantified using Tau (τ)-squared, and heterogeneity was assessed with Cochran’s Q and *I*-squared (*I*^2^), where *I*^2^ < 25% indicated low, 25% to 50% indicated moderate, and > 50% indicated substantial heterogeneity.

### Subgroup and sensitivity analysis

Subgroup and sensitivity analyses were conducted at the class- and agent-level to explore potential sources of heterogeneity. Subgroup analyses included a restriction to participants with established cardiovascular disease or ASCVD where such data were available; due to limited reporting, these analyses were restricted to MACE. Additional subgroup analyses excluded trials using oral semaglutide and short-acting GLP-1RAs, as well as trials with study populations least comparable to SURPASS-CVOT. Leave-one-out analyses were performed to assess the influence of individual trials on pooled effect estimates and heterogeneity.

### Certainty of evidence and risk of bias

Certainty of evidence was assessed using the GRADE approach adapted for NMAs via the Confidence in Network Meta-Analysis (CINeMA) framework [12]. For each comparison in both the class-level and agent-level networks, six domains were evaluated—within-study bias, reporting bias, indirectness, imprecision, heterogeneity, and incoherence—and an overall confidence rating (high, moderate, low, or very low) was assigned. Risk of bias was assessed using the Cochrane Risk of Bias 2 (RoB 2) tool for randomized trials [13]. Each risk of bias and certainty of evidence assessment was independently completed by two reviewers, with discrepancies resolved through consensus.

## Results

**Fig. 2:**
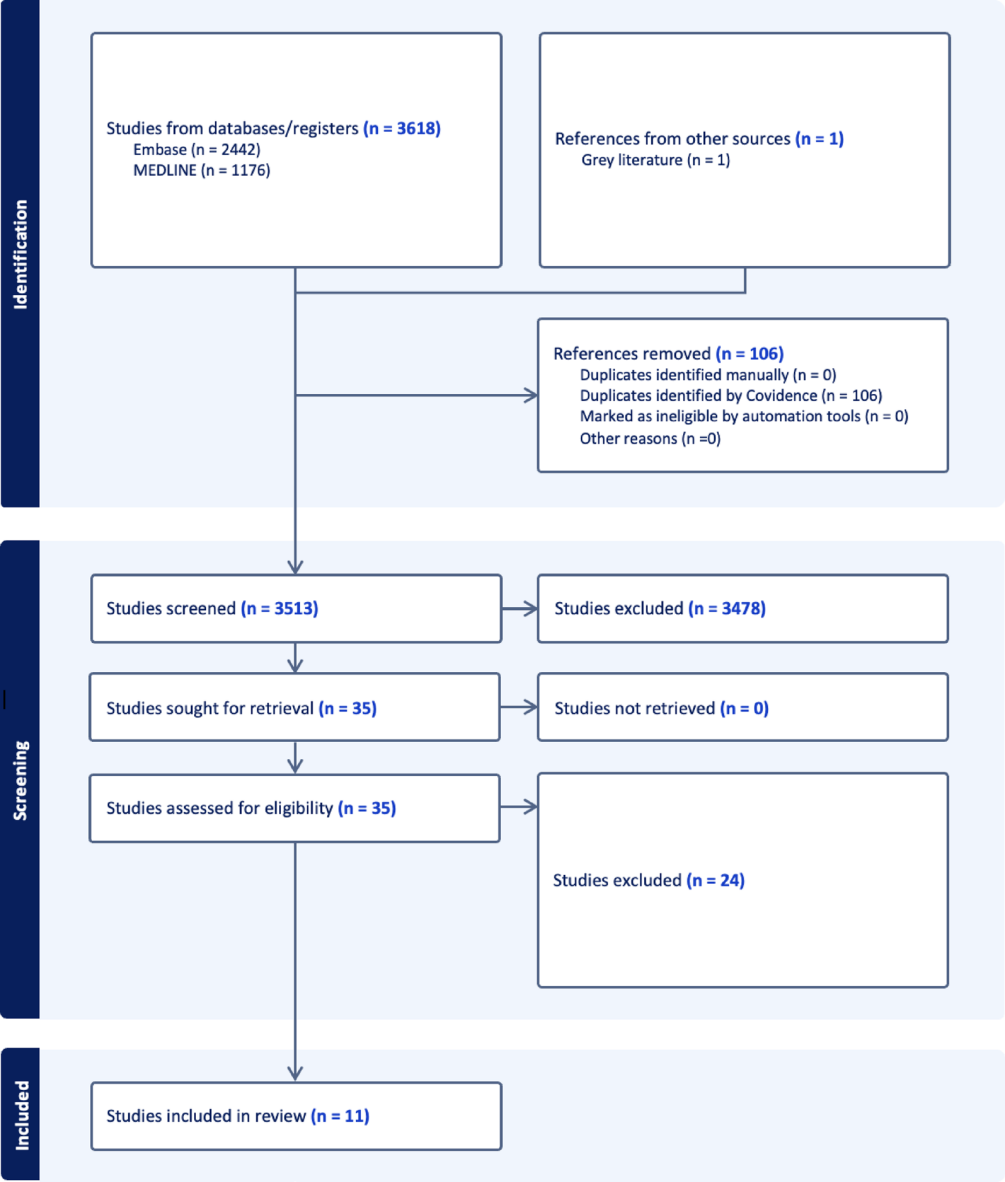
PRISMA flow diagram outlining studying selection process

A total of 3619 studies were identified through searches of MEDLINE, EMBASE, and gray literature. Following removal of 106 duplicates, 3513 studies remained. After title and abstract screening, 35 studies progressed to full-text review (Fig. 2). After full text review, ten trials on GLP-1RAs [14–23] and one on Tirzepatide [24] met inclusion criteria. Baseline information on included trials can be found in Supplementary Table S3.

### Risk of bias results

Risk of bias for the included RCTs was evaluated using the Cochrane RoB 2 tool. All trials were judged to have an overall low risk of bias, with low risk across all individual domains, including the randomization process, deviations from intended interventions, missing outcome data, outcome measurement, and outcome reporting. The RoB summary table can be found in Supplementary Appendix Table S4.

### Class-level network meta-analysis

The class-level NMA evaluated the effect of tirzepatide and the broader GLP-1RA class on cardiovascular outcomes relative to placebo. This framework integrated evidence through the following streams: (1) Tirzepatide compared to placebo (indirect comparison): In the absence of direct tirzepatide CV outcome placebo-controlled trials, these estimates were derived by bridging direct evidence from SURPASS-CVOT with pooled data from multiple GLP-1RA-placebo trials. (2) GLP-1RA Class compared to placebo (direct comparison): This result represents the pooled meta-analytic average of the included GLP-1RA CV outcome trials, establishing a class-wide performance benchmark. (3) Tirzepatide compared to the GLP-1RA class (unable to assess in present NMA): A direct class-level comparison was not formally calculated because the link between tirzepatide and the GLP-1RA class was anchored exclusively to the SURPASS-CVOT trial; because no alternative network pathways or multiple trials existed to inform this node, a pooled class-wide comparison would reflect single-trial evidence rather than a broader class effect.

#### Major adverse cardiovascular events

**Fig. 3:**
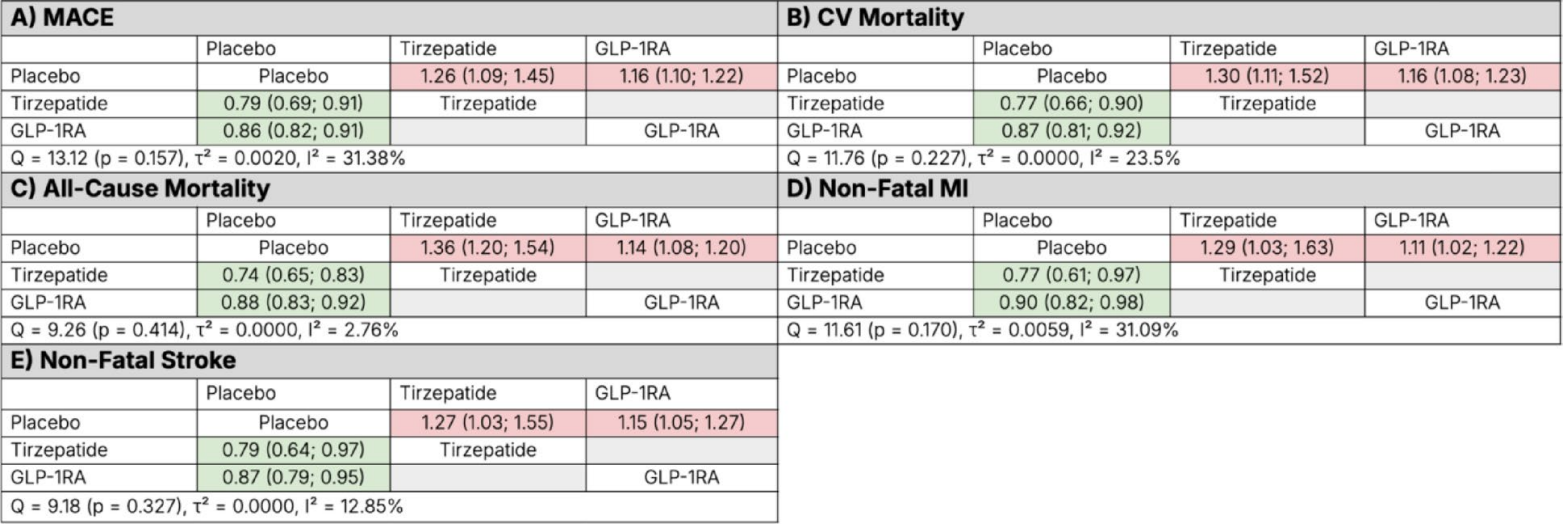
Network meta-analysis league tables comparing tirzepatide and the pooled GLP-1 receptor agonists (GLP-1RAs) to placebo across cardiovascular outcomes: **A** major adverse cardiovascular events (MACE), **B** cardiovascular (CV) mortality, **C** all-cause mortality, **D** non-fatal myocardial infarction (MI), and **E** non-fatal stroke. Hazard ratios with 95% confidence intervals represent the row treatment versus the column treatment. Green shading denotes statistically significant benefit and red shading denotes statistically significant relative harm. Measures of heterogeneity (Q statistic, τ^2^, and I^2^) are provided for each network

**Fig. 4:**
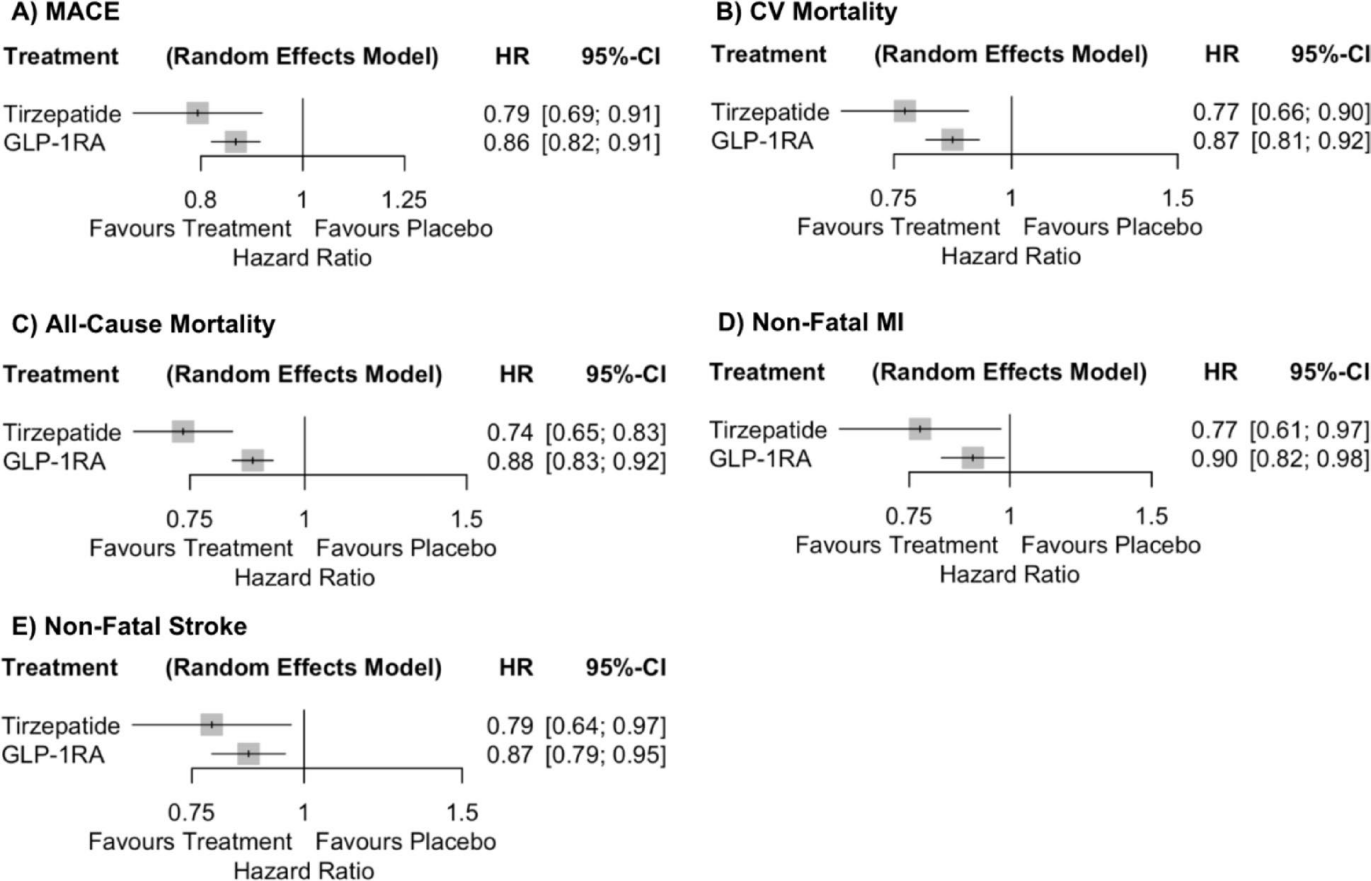
Forest plots from the class-level network meta-analysis comparing tirzepatide, GLP-1RA class, and placebo across cardiovascular outcomes: **A** major adverse cardiovascular events (MACE), **B** cardiovascular (CV) mortality, **C** all-cause mortality, **D** non-fatal myocardial infarction (MI), and **E** non-fatal stroke. Hazard ratios (HRs) with 95% confidence intervals (CIs) are shown for each active treatment versus placebo. Horizontal lines denote 95% CIs; values to the left of the vertical line at HR = 1 favor treatment, while values to the right favor placebo

Compared with placebo, tirzepatide was associated with a significant reduction in MACE (HR 0.79, 95% CI 0.69–0.91). Compared with placebo, the GLP-1RA class was associated with a significant reduction in MACE (HR 0.86, 95% CI 0.82–0.91). Between-study heterogeneity was moderate (Q = 13.12, *p* = 0.157; τ^2^ = 0.0020; I = 31.4%) (Fig. 3, Panel A). Figure 4, Panel A, presents a forest plot of HRs for MACE, illustrating a numerically lower point estimate for tirzepatide compared to the GLP-1RA class, with overlapping CIs and no formal statistical comparison.

#### Cardiovascular mortality

Compared with placebo, tirzepatide and the GLP-1RA class significantly reduced the risk of CV mortality (HR 0.77, 95% CI 0.66–0.90 and HR 0.87, 95% CI 0.81–0.92, respectively).- Heterogeneity across studies was low (Q = 11.76, *p* = 0.227; τ^2^ = 0.0000; I^2^ = 23.5%) (Fig. 3, Panel B). Figure 4, Panel B, presents a forest plot of HRs for CV mortality, illustrating a numerically lower point estimate for tirzepatide compared to the GLP-1RA class.

#### All-cause mortality

Compared with placebo, tirzepatide and the GLP-1RA class significantly reduced the risk of all-cause mortality (HR 0.74, 95% CI 0.65–0.83 and HR 0.88, 95% CI 0.83–0.92, respectively). Heterogeneity was low (Q = 9.26, *p* = 0.414; τ^2^ = 0.0000; I^2^ = 2.8%) (Fig. 3, Panel C). Figure 4, Panel C, presents a forest plot of HRs for all-cause mortality, illustrating a numerically lower point estimate for tirzepatide compared to the GLP-1RA class.

#### Non-fatal myocardial infarction

Compared with placebo, tirzepatide and the GLP-1RA class significantly reduced the risk of non-fatal MI (HR 0.77, 95% CI 0.61–0.97 and HR 0.90, 95% CI 0.82–0.98, respectively). Between-study heterogeneity was moderate (Q = 11.61, *p* = 0.170; τ^2^ = 0.0059; I^2^ = 31.1%) (Fig. 3, Panel D). Figure 4, Panel D, presents a forest plot of HRs for non-fatal MI, illustrating a numerically lower point estimate for tirzepatide compared to the GLP-1RA class.

#### Non-fatal stroke

Compared with placebo, tirzepatide and the GLP-1RA class significantly reduced the risk of non-fatal stroke (HR 0.79, 95% CI 0.64–0.97 and HR 0.87, 95% CI 0.79–0.95, respectively). Heterogeneity was low (Q = 9.18, *p* = 0.327; τ^2^ = 0.0000; I^2^ = 12.9%) (Fig. 3, Panel E). Figure 4, Panel E, presents a forest plot of HRs for non-fatal stroke, illustrating a numerically lower point estimate for tirzepatide compared to the GLP-1RA class.

### Agent-level network meta-analysis

#### Major adverse cardiovascular events

**Fig. 5:**
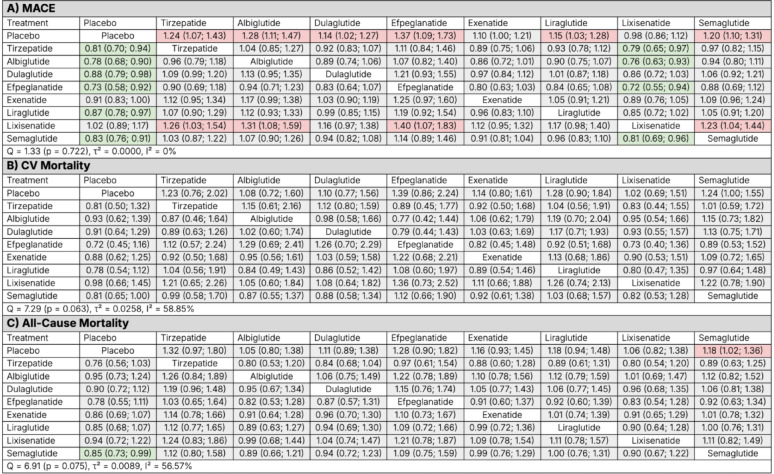
Network meta-analysis league tables comparing tirzepatide, individual GLP-1 receptor agonists, and placebo for **A** major adverse cardiovascular events (MACE), **B** cardiovascular (CV) mortality, and **C** all-cause mortality. Hazard ratios (HRs) with 95% confidence intervals represent the row treatment versus the column treatment. Green shading denotes statistically significant benefit, red shading denotes statistically significant relative harm, and gray indicates no statistically significant difference. Heterogeneity estimates (Q statistic, τ^2^, and I^2^) are provided for each network

**Fig. 6:**
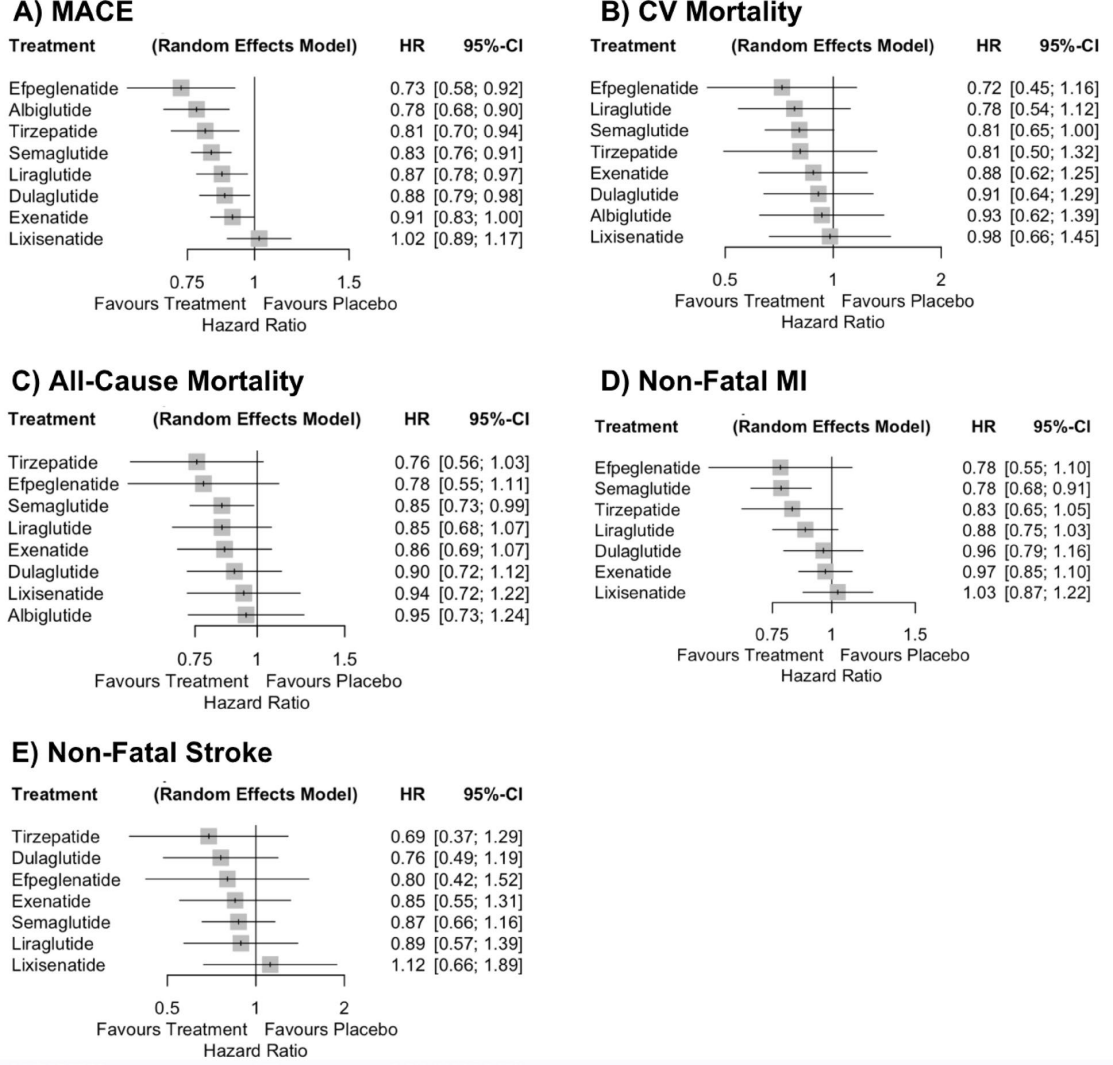
Forest plots from the agent-level network meta-analysis comparing tirzepatide, individual GLP-1 receptor agonists, and placebo across cardiovascular outcomes: **A** major adverse cardiovascular events (MACE), **B** cardiovascular (CV) mortality, **C** all-cause mortality, **D** non-fatal myocardial infarction (MI), and **E** non-fatal stroke. Hazard ratios (HRs) with 95% confidence intervals (CIs) are shown for each active treatment versus placebo. Horizontal lines denote 95% CIs; values to the left of the vertical line at HR = 1 favor treatment, while values to the right favor placebo

In the agent-level NMA, tirzepatide significantly reduced the risk of MACE compared with placebo (HR 0.81, 95% CI 0.70–0.94) and with lixisenatide (HR 0.79, 95% CI 0.65–0.97). For all other agents including albiglutide, dulaglutide, efpeglenatide, exenatide, liraglutide, and semaglutide, tirzepatide showed no statistically significant differences in MACE risk. Between-study heterogeneity was negligible (Q = 1.33, *p* = 0.722; τ^2^ = 0.0000; I^2^ = 0%) (Fig. 5, Panel A). Figure 6, Panel A, presents a forest plot of HRs ranked from the greatest to least reduction in MACE.

#### Cardiovascular mortality

Tirzepatide did not significantly reduce CV mortality compared with placebo (HR 0.81, 95% CI 0.50–1.32). No statistically significant differences were observed between tirzepatide and any other agent. For all other comparators there were no significant differences in CV mortality risk. Between-study heterogeneity was high (Q = 7.29, *p* = 0.063; τ^2^ = 0.0258; I^2^ = 58.9%) (Fig. 5, Panel B). Figure 6, Panel B, presents a forest plot of HRs ranked from the greatest to least reduction in CV mortality.

#### All-cause mortality

Tirzepatide showed a non-significant trend toward reduction in all-cause mortality compared with placebo (HR 0.76, 95% CI 0.56–1.03), whereas semaglutide was the only agent that achieved a statistically significant reduction (HR 0.85, 95% CI 0.73–0.99). No statistically significant differences were observed between tirzepatide and any other agent. For all other comparators there were no significant differences in all-cause mortality risk. Between-study heterogeneity was substantial (Q = 6.91, *p* = 0.075; τ^2^ = 0.0089; I^2^ = 56.6%) (Fig. 5, Panel C). Figure 6, Panel C, presents a forest plot of HRs ranked from the greatest to least reduction in all-cause mortality.

#### Non-fatal myocardial infarction

**Fig. 7:**
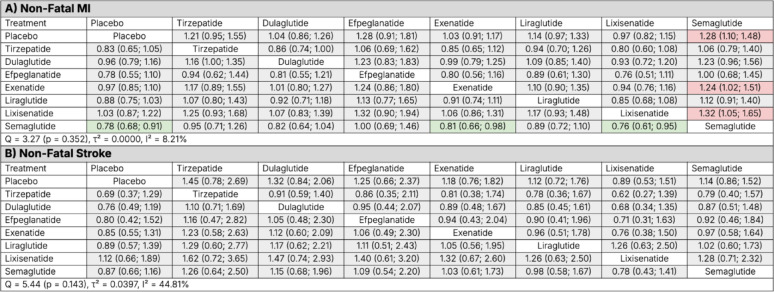
Network meta-analysis league tables comparing tirzepatide, individual GLP-1 receptor agonists, and placebo for **A** non-fatal myocardial infarction (MI) and **B** non-fatal stroke. Hazard ratios (HRs) with 95% confidence intervals represent the row treatment versus the column treatment. Green shading denotes statistically significant benefit, red shading denotes statistically significant relative harm, and gray indicates no statistically significant difference. Heterogeneity estimates (Q statistic, τ^2^, and I^2^) are provided for each network

Tirzepatide showed a non-significant trend toward reduction in non-fatal MI compared with placebo (HR 0.83, 95% CI 0.65–1.05), whereas semaglutide was the only agent that achieved a statistically significant reduction (HR 0.78, 95% CI 0.68–0.91). No statistically significant differences were observed between tirzepatide and any other agent. Between-study heterogeneity was low (Q = 3.27, *p* = 0.352; τ^2^ = 0.0000; I^2^ = 8.2%) (Fig. 7, Panel A). Figure 6, Panel D, presents a forest plot of HRs ranked from the greatest to least reduction in non-fatal MI.

#### Non-fatal stroke

Tirzepatide showed a non-significant trend toward reduction in non-fatal stroke compared with placebo (HR 0.69, 95% CI 0.37–1.29). For all other comparators there were no significant differences in stroke risk (HRs ranged from 0.78 to 1.29). Between-study heterogeneity was moderate (Q = 5.44, *p* = 0.143; τ^2^ = 0.0397; I^2^ = 44.8%) (Fig. 7, Panel B). Figure 6, Panel E, presents a forest plot of HRs ranked from the greatest to least reduction in non-fatal stroke.

### Subgroup and sensitivity analyses

To explore potential sources of heterogeneity, three subgroup analyses were conducted at the class- and agent-level.

The first subgroup included studies enrolling participants with established cardiovascular disease or providing cardiovascular disease or ASCVD-restricted subgroup data. At the class-level, estimates for MACE and measures of heterogeneity were consistent with the overall analysis (Supplementary Figure S1). Compared with placebo, both tirzepatide (HR 0.78, 95% CI 0.66–0.93) and the GLP-1RA class (HR 0.85, 95% CI 0.80–0.91) were associated with lower risk of MACE, with moderate heterogeneity (Q = 13.9 [*p* = 0.126], τ^2^ = 0.0040, I^2^ = 35.26%). At the agent level, in the established cardiovascular disease subgroup analysis, tirzepatide was associated with a larger relative point-estimate reduction in MACE versus placebo (HR 0.73 [0.59–0.90]) (Supplementary Figure S2) compared with the broader network (HR 0.81 [0.70–0.94]) (Fig. 5). Compared to placebo, GLP-1RA agents generally showed numerically lower MACE risk in the established cardiovascular disease subgroup (including dulaglutide, efpeglenatide, exenatide, and liraglutide), with the exception of semaglutide; however, these observations do not represent a formal statistical comparison. Between-study heterogeneity within the established cardiovascular disease network was negligible (Q = 2.31 [*p* = 0.511], τ^2^ = 0.0000, I^2^ = 0%).

Additionally, a class-level subgroup analysis restricted to injectable long-acting agents was conducted by excluding trials of oral semaglutide (PIONEER 6 and SOUL) and the short-acting lixisenatide trial (ELIXA) at the class-level. A separate class-level subgroup analysis excluding trials with study populations least comparable to SURPASS-CVOT, specifically ELIXA, which enrolled patients with recent acute coronary syndrome (within 180 days of medication initiation), and FLOW, a dedicated CKD outcomes trial. Across these subgroup analyses, findings were consistent with the primary class-level NMA. When restricting to injectable long-acting agents, heterogeneity decreased for most outcomes (MACE, CV mortality, all-cause mortality, and non-fatal MI), although the magnitude of reduction varied and was not directionally consistent across all outcomes (Supplementary Figure S3). Likewise, excluding trials least comparable to SURPASS-CVOT produced concordant effect estimates and did not consistently decrease heterogeneity across outcomes (Supplementary Figure S4).

In leave-one-out sensitivity analysis at the class-level, the trials most frequently contributing to between-study heterogeneity were ELIXA, FLOW, and PIONEER-6, with SOUL and SUSTAIN-6 occasionally identified as additional influential contributors depending on the outcome. However, sequential exclusion of these trials resulted in only modest changes in pooled HRs and corresponding CIs, with no substantial shifts in the direction of effect, significance, or overall interpretation of the primary findings (Supplementary Figures S5–S9). Similar findings were seen at the agent-level (Supplementary Table S5–S24).

### Certainty of evidence

At the class-level, certainty of evidence was highest for the GLP-1RA class compared with placebo, with high certainty observed for CV mortality, all-cause mortality, and non-fatal stroke, and moderate certainty for MACE and non-fatal MI, secondary to heterogeneity or imprecision for certain outcomes (Supplementary Table S25). In contrast, certainty of evidence for tirzepatide compared to placebo at the agent-level was consistently low across outcomes, primarily driven by indirect-only evidence and concerns related to transitivity, with additional downgrading for imprecision or heterogeneity in select endpoints (Supplementary Table S25).

At the agent-level, several individual GLP-1RAs demonstrated moderate to high certainty for MACE when compared with placebo, whereas certainty for other outcomes was generally low to moderate, reflecting wider confidence intervals and greater between-study heterogeneity (Supplementary Table S26). Across outcomes, agent-level estimates for tirzepatide compared with placebo were predominantly low to very low certainty, driven by indirect-only evidence and imprecision. Most tirzepatide compared to individual GLP-1RAs were similarly rated very low as they were informed exclusively by indirect evidence (Supplementary Table S26).

## Discussion

In this review, the class-level NMA showed that tirzepatide was associated with significant reductions in MACE, all-cause mortality, CV mortality, non-fatal MI, and non-fatal stroke compared to placebo in patients with T2D and ASCVD or high CV risk. However, in the agent-level analysis, tirzepatide only demonstrated significant reductions in MACE when compared to placebo, while other endpoints showed favorable but non-significant trends.

Given the limitations of the evidence and the constraints of a class-level NMA, a pooled comparison between tirzepatide and the GLP-1RA class was not possible. However in the class-level NMA, tirzepatide consistently demonstrated numerically lower point estimates than the GLP-1RA class across all outcomes when compared to placebo. In the agent-level analysis, tirzepatide demonstrated comparable efficacy to the majority of individual GLP-1RAs across outcomes. These findings suggest that tirzepatide confers cardiovascular benefits at least comparable to established GLP-1RAs. While class-level point estimates favor tirzepatide, these findings do not reflect formal statistical comparison and should be interpreted cautiously as hypothesis-generating; accordingly, additional head-to-head trials are required to define tirzepatide’s cardiovascular risk reduction benefit compared to other GLP-1RAs, particularly semaglutide, the most widely used GLP-1RA in contemporary practice.

Although both the class- and agent-level analyses were derived from the same RCTs, their results differed which is most apparent when looking at the tirzepatide versus placebo data. The class-level analysis pooled all GLP-1RAs into a single node, increasing statistical power and yielding narrower CIs, but at the cost of assuming similar effects across individual agents. Pooling strengthened indirect evidence and narrowed variance, but likely masked agent-specific differences within the GLP-1RA class. Importantly, GLP-1RAs are not identical; short-acting exendin-based agents such as lixisenatide have distinct pharmacokinetic profiles from long-acting analogues such as semaglutide or dulaglutide, which translate into differences in efficacy and side-effect profiles [25]. Moreover, even among long-acting formulations, efficacy varies according to agent, dose, and route of administration [26, 27]. In the exploratory subgroup analyses, effect estimates remained directionally consistent with the primary analysis across all subgroups, with heterogeneity decreasing for some, but not all, outcomes. In the agent-level analysis, evidence for most GLP-1RAs—except semaglutide—was derived from a single trial. Given the limited evidence base, statistical comparisons of individual GLP-1RAs compared to placebo were confined to the findings of their respective trials. Additionally, given the limited data, precision was reduced, confidence intervals widened, and statistical significance was often lost. Ultimately, class-level estimates may overgeneralize or oversimplify benefits, whereas agent-level estimates may understate class-wide effects due to sparse data for individual agents. Nevertheless, several large observational studies have suggested possible incremental advantages of tirzepatide over GLP-1RAs for cardiovascular and renal outcomes, an effect that is biologically plausible through its dual GIP/GLP-1 agonism and further reinforced by greater bodyweight reduction and improved glycemic control [8].

In a real-world retrospective observational cohort of more than 140,000 patients with T2D, tirzepatide was associated with greater 0.34 percentage point reduction in glycated hemoglobin A1c (HbA1c), 2.9 kg body weight reduction, in addition to lower rates of all-cause mortality (HR 0.58, 95% CI 0.45–0.75), MACE (HR 0.80, 95% CI 0.71–0.91), and major adverse kidney events (HR 0.54, 95% CI 0.44–0.67) compared with GLP-1RAs [28]. In another observational study of patients aged ≥ 40 years with T2D, body mass index ≥ 25 kg/m^2^, and pre-existing ischemic heart disease, tirzepatide was associated with a lower rate of the primary composite outcome of all-cause mortality, myocardial infarction, and ischemic stroke (HR 0.60, 95% CI 0.43–0.84), as well as reductions in all individual components of the composite outcome [29]. In another recent observational trial, tirzepatide was associated with lower MACE risk, compared to semaglutide (HR 0.86, 95% CI 0.74–0.99) and liraglutide (HR 0.58, 95% CI 0.51–0.66) in patients with T2D and obstructive sleep apnea [30].

Semaglutide is the most extensively studied GLP-1RA, with multiple trials, including SUSTAIN-6, PIONEER-6, FLOW, and SOUL [20–22, 31], collectively establishing a robust evidence base for cardiorenal risk reduction in T2D, with SELECT extending these benefits to adults with overweight/obesity and established ASCVD without diabetes [32]. In our agent-level analysis, tirzepatide and semaglutide showed no statistically significant differences across outcomes. Comparative real-world studies have reported mixed findings. Data from STEER [33] suggests lower incidence of 3-point MACE with semaglutide compared to tirzepatide in patients with overweight/obesity and ASCVD without diabetes. Given its retrospective, claims-based design, this study is susceptible to residual confounding and outcome misclassification. In addition, the study had a short follow-up duration, and thus findings should be interpreted as hypothesis-generating. In contrast, recent trial-emulation and observational studies have reported broadly comparable or superior cardiovascular risk reduction for tirzepatide compared to semaglutide [30, 34]. Collectively, these discordant observational findings highlight the need for randomized head-to-head trials to clarify relative effects on CV outcomes between tirzepatide and semaglutide.

## Future directions

Future research should explore combination strategies, including dual and triple therapy regimens pairing tirzepatide with other agents including sodium-glucose cotransporter-2 inhibitors and the non-steroidal mineralocorticoid receptor antagonist, finerenone, which have been shown to offer additive cardiorenal protection alongside GLP-1RAs [35]. Beyond tirzepatide, other incretin-based therapies have shown promise, such as retatrutide, a novel triple-hormone receptor agonist that simultaneously activates GLP-1, GIP, and glucagon receptors, with early-phase trials demonstrating profound effects on weight reduction [36]. Several additional tirzepatide trials are ongoing, including SURMOUNT-MMO, which is evaluating whether the cardiovascular benefits of tirzepatide extend to individuals with overweight or obesity without diabetes (37).

## Limitations

This study has several limitations that warrant consideration. First, the certainty of evidence for tirzepatide outcomes was low or very low as estimates were driven largely by indirect evidence, with additional downgrading for imprecision and heterogeneity for select outcomes. Notably, only a single trial evaluated tirzepatide, and its position within the treatment network relied on indirect connection via dulaglutide to the broader GLP-1RA evidence base, which limits the strength of comparative inference.

Next, the agent-level analysis was constrained by the limited number of trials available for most GLP-1RAs, with several agents represented by only a single study. In contrast, the class-level analysis assumes homogeneity across all GLP-1RAs, an assumption that does not always hold given population and pharmacologic differences across studies. Importantly, clinically meaningful differences exist even among long-acting GLP-1RAs, including variations in molecular structure, dosing, and formulation, which may translate into differential cardiovascular effects.

NMAs are subject to inherent limitations, including assumptions of transitivity and consistency, which are not always met due to heterogeneity across trials. To address potential sources of heterogeneity and intransitivity, we conducted multiple subgroup analyses, including restriction to established cardiovascular disease cohorts. While analyses restricted to participants with established cardiovascular disease yielded MACE results that were directionally consistent with the overall analysis, conclusions are limited because subgroup estimates were based on trial-level reporting rather than individual participant data, and included studies continued to differ in patient characteristics, definitions of established cardiovascular disease, background therapies, and trial era. These factors likely contribute to the persistence of moderate heterogeneity observed in the subgroup analysis. Additionally, subgroup and sensitivity analyses were not prespecified and therefore may be subject to bias.

Accordingly, although our results suggest tirzepatide confers cardiovascular benefits at least comparable to the GLP-1RA class, confirmation from adequately powered randomized head-to-head cardiovascular outcome trials will be important to define the magnitude of benefit.

## Conclusion

In this network meta-analysis, tirzepatide was associated with significant reductions in MACE, CV mortality, all-cause mortality, non-fatal MI, and non-fatal stroke compared with placebo in the class-level analysis. Formal statistical comparisons between tirzepatide and the GLP-1RA class could not be performed within the constraints of the NMA; however, point estimates consistently favored tirzepatide compared with placebo. At the agent-level, tirzepatide demonstrated generally comparable efficacy to individual GLP-1RA agents. Interpreted alongside observational data and mechanistic evidence, these findings support tirzepatide as an effective therapy for cardiovascular risk reduction in patients with T2D and established ASCVD or high CV risk, with benefits at least comparable to GLP-1RAs. Nevertheless, given residual heterogeneity and the inherent limitations of network meta-analyses, further randomized trials are needed to confirm these findings, define relative effects versus individual GLP-1RAs, and clarify the role of tirzepatide across broader cardiometabolic populations.

## Supplementary Information

Below is the link to the electronic supplementary material.

Supplementary Material 1

## Data Availability

No new, previously unpublished patient data were generated or analyzed in support of this research.
