# Supplementary material for: Comparative efficacy of tirzepatide and glucagon-like peptide-1 receptor agonists on cardiovascular outcomes in patients with type 2 diabetes: a systematic review and network meta-analysis
Source: Cardiovasc Diabetol. 2026 Feb 27;25:115. doi: 10.1186/s12933-026-03113-3 (PMC13049956; doi:10.1186/s12933-026-03113-3)
Supplement: Supplementary file 1 — Supplementary Material 1 [file 12933_2026_3113_MOESM1_ESM.pdf]

Supplementary Appendix: Comparative Efficacy of Tirzepatide and Glucagon-Like Peptide-1 Receptor Agonists on Cardiovascular Outcomes in Patients with Type 2 Diabetes: A Systematic Review and Network Meta-Analysis

Search Strategy

MEDLINE:

#	Search Strategy	Results
1	tirzepatide.mp. or exp Tirzepatide/	1,318
2	GLP-1 receptor agonist.mp. or exp Glucagon-Like Peptide-1 Receptor Agonists/	10,207
3	albiglutide.mp.	265
4	dulaglutide.mp.	1,079
5	efpeglenatide.mp.	47
6	exenatide.mp. or exp Exenatide/	4,232
7	liraglutide.mp. or exp Liraglutide/	5,196
8	lixisenatide.mp.	685
9	semaglutide.mp.	3,534
10	type 2 diabetes.mp. or exp Diabetes Mellitus, Type 2/	264,952
11	1 or 2 or 3 or 4 or 5 or 6 or 7 or 8 or 9	15,229
12	10 and 11	8,852
13	randomized controlled trial.mp. or exp Randomized Controlled Trial/	708,224
14	12 and 13	1,213
15	limit 14 to (english language and humans)	1,176

Supplemental Table S1. MEDLINE search strategy.

Embase:

#	Search Strategy	Results
1	tirzepatide.mp. or exp tirzepatide/	3,180
2	GLP-1 receptor agonist.mp. or exp glucagon like peptide 1 receptor agonist/	67,938
3	albiglutide.mp. or exp albiglutide/	1,804
4	dulaglutide.mp. or exp dulaglutide/	4,482
5	efpeglenatide.mp. or exp efpeglenatide/	275
6	exenatide.mp.	5,365
7	exp liraglutide/ or liraglutide.mp.	17,299
8	exp lixisenatide/ or lixisenatide.mp.	3,069
9	semaglutide.mp. or exp semaglutide/	9,943
10	type 2 diabetes.mp. or exp non insulin dependent diabetes mellitus/	482,658
11	1 or 2 or 3 or 4 or 5 or 6 or 7 or 8 or 9	68,510
12	10 and 11	36,064
13	randomized controlled trial.mp. or exp randomized controlled trial/	1,424,267
14	12 and 13	7,027
15	limit 14 to (human and english language and "remove clinical trial (clinicaltrials.gov) records" and "remove medline records")	2,442

Supplemental Table S2. Embase search strategy.

Baseline Table

Trial	Intervention versus Comparator	Title	Population	Sample Size (N)	Age [mean (SD)]
SURPASS-CVOT	Tirzepatide vs Dulaglutide	Comparison of tirzepatide and dulaglutide on major adverse cardiovascular events in participants with type 2 diabetes and atherosclerotic cardiovascular disease: SURPASS-CVOT	T2D with established ASCVD, and BMI ≥25 kg/m²	Tirzepatide: 6586<br>Dulaglutide: 6579	Tirzepatide: 64.0<br>Dulaglutide: 64.1
AMPLITUDE-O	Efpeglenatide vs Placebo	Cardiovascular and Renal Outcomes with Efpeglenatide in Type 2 Diabetes	T2D and either a history of CV or kidney disease and at least one other CV risk factor	I: 2717<br>C: 1359	I: 64.6±8.2<br>C: 64.4±8.3
ELIXA	Lixisenatide vs Placebo	Lixisenatide in Patients with Type 2 Diabetes and Acute Coronary Syndrome	T2D with MI or hospitalization for unstable angina within past 180 days	I: 3034<br>C: 3034	I: 59.9±9.7<br>C: 60.6±9.6
EXSCEL	Exenatide vs Placebo	Effects of Once-Weekly Exenatide on Cardiovascular Outcomes in Type 2 Diabetes	T2D with established CV disease or CV risk factors	I: 7356<br>C: 7396	I: 62.0 (range: 56-68)<br>C: 62 (range: 56-68
FLOW	Semaglutide vs Placebo	Effects of Semaglutide on Chronic Kidney Disease in Patients with Type 2 Diabetes	T2D and CKD	I: 1767<br>C: 1766	I: 66.6±9.0<br>C: 66.7±9.0
Harmony Outcomes	Albiglutide vs Placebo	Albiglutide and cardiovascular outcomes in patients with type 2 diabetes and cardiovascular disease (Harmony Outcomes): a double-blind, randomised placebo-controlled trial	T2D and established ASCVD	I: 4731<br>C: 4732	I: 64.1±8.7<br>C: 64.2±8.7

LEADER	Liraglutide vs Placebo	Liraglutide and Cardiovascular Outcomes in Type 2 Diabetes	T2D and established CV disease or high CV risk	I: 4668<br>C: 4672	I: 64.2±7.2<br>C: 64.4±7.2
PIONEER 6	Oral Semaglutide vs Placebo	Oral Semaglutide and Cardiovascular Outcomes in Patients with Type 2 Diabetes	T2D and established CV disease or CKD or high CV risk	I: 1591<br>C: 1592	I: 66±7<br>C: 66±7
SOUL	Oral Semaglutide vs Placebo	Oral Semaglutide and Cardiovascular Outcomes in High-Risk Type 2 Diabetes	T2D with ASCVD, CKD, or both	4,825<br>4,825	I: 66.1±7.6<br>C: 66.1±7.5
SUSTAIN 6	Semaglutide vs Placebo	Semaglutide and Cardiovascular Outcomes in Patients with Type 2 Diabetes	T2D with established CV disease or high CV risk	I: male 1013, female 635<br>C: male 989, female 660	I: male 64.6±7.3, female 64.8±7.1<br>C: male 64.6±7.6, female 64.6±7.5
REWIND	Dulaglutide vs Placebo	Dulaglutide and cardiovascular outcomes in type 2 diabetes (REWIND): a double-blind, randomised placebo-controlled trial	T2D with previous CV event or high CV risk	I: 4949<br>C: 4952	I: 66.2±6.5<br>C: 66.2±6.5

Supplemental Table S3. Summary of major cardiovascular and renal outcome trials of GLP-1RAs and tirzepatide in patients with T2D and ASCVD or high CV risk. The table outlines the study name, intervention versus (vs) comparator, enrolled population, and sample size (N). I = intervention group; C = control group.

Risk of Bias

Study, Year	Randomization Process	Intervention Deviation	Missing Outcome Data	Measurement of the Outcome	Reporting of the Outcome	Overall Risk
AMPLITUDE-O, 2021	Low Risk	Low Risk	Low Risk	Low Risk	Low Risk	Low Risk
ELIXA, 2015	Low Risk	Low Risk	Low Risk	Low Risk	Low Risk	Low Risk
EXSCEL, 2017	Low Risk	Low Risk	Low Risk	Low Risk	Low Risk	Low Risk

FLOW, 2024	Low Risk	Low Risk	Low Risk	Low Risk	Low Risk	Low Risk
Harmony Outcomes, 2018	Low Risk	Low Risk	Low Risk	Low Risk	Low Risk	Low Risk
LEADER, 2016	Low Risk	Low Risk	Low Risk	Low Risk	Low Risk	Low Risk
PIONEER 6, 2019	Low Risk	Low Risk	Low Risk	Low Risk	Low Risk	Low Risk
SOUL, 2025	Low Risk	Low Risk	Low Risk	Low Risk	Low Risk	Low Risk
SUSTAIN 6, 2016	Low Risk	Low Risk	Low Risk	Low Risk	Low Risk	Low Risk
REWIND, 2019	Low Risk	Low Risk	Low Risk	Low Risk	Low Risk	Low Risk

Supplemental Table S4. Risk of bias assessment for included randomized controlled trials. The Cochrane Risk of Bias 2 tool was used to assess methodological quality across five domains: randomization process, deviations from intended interventions, missing outcome data, measurement of the outcome, and selection of the reported result.

Subgroup Analysis

**MACE**			
	Placebo	Tirzepatide	GLP-1RA
Placebo	Placebo	1.28 (1.07; 1.52)	1.17 (1.10; 1.25)
Tirzepatide	0.78 (0.66; 0.93)	Tirzepatide	
GLP-1RA	0.85 (0.80; 0.91)		GLP-1RA
Q = 13.9 (p = 0.126), τ^2 = 0.0040, I^2 = 35.26%			

Supplemental Figure S1. Class-level network meta-analysis comparing tirzepatide and the GLP-1RA class to placebo for major adverse cardiovascular events (MACE) restricted to participants with established atherosclerotic cardiovascular disease (ASCVD) or cardiovascular disease (CVD). Hazard ratios (HRs) with 95% confidence intervals represent the row treatment versus the column treatment. Green shading denotes statistically significant benefit, red shading denotes statistically significant relative harm. Values less than 1 favor the active treatment, while values greater than 1 favor the comparator. Measures of between-study heterogeneity (Q statistic, τ^2, and I^2) are reported. This analysis included data from randomized cardiovascular outcomes trials reporting outcomes specifically among participants with established ASCVD, including SURPASS-CVOT, SOUL, LEADER, SUSTAIN-6, PIONEER-6, REWIND, EXSCEL, AMPLITUDE-O, HARMONY Outcomes, and ELIXA. For trials enrolling mixed primary and

secondary prevention populations, hazard ratios derived from prespecified or post-hoc subgroup analyses restricted to participants with established ASCVD or CVD were used. Of note, the trial-defined "established CVD" subgroups in LEADER, SUSTAIN-6, and PIONEER 6 included participants enrolled on the basis of chronic kidney disease alone (i.e., without established ASCVD), in accordance with each study's predefined subgroup definitions.

**MACE**									
Treatment	Placebo	Tirzepatide	Albiglutide	Dulaglutide	Efpeglanatide	Exenatide	Liraglutide	Lixisenatide	Semaglutide
Placebo	Placebo	1.38 (1.11; 1.70)	1.28 (1.11; 1.47)	1.27 (1.05; 1.53)	1.39 (1.07; 1.81)	1.11 (1.01; 1.23)	1.20 (1.07; 1.35)	0.98 (0.86; 1.12)	1.17 (1.06; 1.30)
Tirzepatide	0.73 (0.59; 0.90)	Tirzepatide	0.93 (0.72; 1.20)	0.92 (0.83; 1.01)	1.01 (0.72; 1.41)	0.81 (0.64; 1.02)	0.88 (0.69; 1.11)	0.71 (0.55; 0.92)	0.85 (0.67; 1.08)
Albiglutide	0.78 (0.68; 0.90)	1.07 (0.83; 1.38)	Albiglutide	0.99 (0.78; 1.25)	1.08 (0.80; 1.46)	0.87 (0.73; 1.03)	0.94 (0.78; 1.13)	0.76 (0.63; 0.93)	0.91 (0.77; 1.09)
Dulaglutide	0.79 (0.66; 0.95)	1.09 (0.99; 1.20)	1.01 (0.80; 1.28)	Dulaglutide	1.10 (0.79; 1.51)	0.88 (0.71; 1.09)	0.95 (0.76; 1.19)	0.77 (0.61; 0.98)	0.93 (0.75; 1.15)
Efpeglanatide	0.72 (0.55; 0.94)	0.99 (0.71; 1.39)	0.92 (0.69; 1.24)	0.91 (0.66; 1.26)	Efpeglanatide	0.80 (0.60; 1.06)	0.87 (0.65; 1.16)	0.71 (0.52; 0.95)	0.84 (0.64; 1.12)
Exenatide	0.90 (0.81; 0.99)	1.24 (0.98; 1.56)	1.15 (0.97; 1.37)	1.14 (0.92; 1.41)	1.25 (0.94; 1.66)	Exenatide	1.08 (0.93; 1.26)	0.88 (0.75; 1.04)	1.06 (0.92; 1.22)
Liraglutide	0.83 (0.74; 0.93)	1.14 (0.90; 1.45)	1.06 (0.89; 1.28)	1.05 (0.84; 1.31)	1.15 (0.87; 1.54)	0.92 (0.79; 1.07)	Liraglutide	0.81 (0.68; 0.97)	0.97 (0.84; 1.13)
Lixisenatide	1.02 (0.89; 1.17)	1.40 (1.09; 1.81)	1.31 (1.08; 1.59)	1.29 (1.02; 1.63)	1.42 (1.05; 1.90)	1.13 (0.96; 1.34)	1.23 (1.03; 1.47)	Lixisenatide	1.20 (1.01; 1.42)
Semaglutide	0.85 (0.77; 0.94)	1.17 (0.93; 1.48)	1.09 (0.92; 1.30)	1.08 (0.87; 1.34)	1.18 (0.89; 1.57)	0.95 (0.82; 1.09)	1.03 (0.88; 1.20)	0.84 (0.70; 0.99)	Semaglutide
$Q = 2.31$ ($p = 0.511$), $\tau^2 = 0.0000$, $I^2 = 0\%$									

Supplemental Figure S2. Agent-level network meta-analysis comparing tirzepatide and the individual GLP-1RA agent to placebo for major adverse cardiovascular events (MACE) restricted to participants with established atherosclerotic cardiovascular disease (ASCVD) or cardiovascular disease (CVD). Hazard ratios (HRs) with 95% confidence intervals represent the row treatment versus the column treatment. Green shading denotes statistically significant benefit, red shading denotes statistically significant relative harm. Values less than 1 favor the active treatment, while values greater than 1 favor the comparator. Measures of between-study heterogeneity (Q statistic, τ^2, and I^2) are reported. This analysis included data from randomized cardiovascular outcomes trials reporting outcomes specifically among participants with established ASCVD, including SURPASS-CVOT, SOUL, LEADER, SUSTAIN-6, PIONEER-6, REWIND, EXSCEL, AMPLITUDE-O, HARMONY Outcomes, and ELIXA. For trials enrolling mixed primary and secondary prevention populations, hazard ratios derived from prespecified or post-hoc subgroup analyses restricted to participants with established ASCVD or CVD were used. Of note, the trial-defined "established CVD" subgroups in LEADER, SUSTAIN-6, and PIONEER 6 included participants enrolled on the basis of chronic kidney disease alone (i.e., without established ASCVD), in accordance with each study's predefined subgroup definitions.

**A) MACE**			
	Placebo	Tirzepatide	GLP-1RA
Placebo	Placebo	1.28 (1.13; 1.45)	1.18 (1.11; 1.24)
Tirzepatide	0.78 (0.69; 0.89)	Tirzepatide	
GLP-1RA	0.85 (0.80; 0.90)		GLP-1RA
Q = 7.06 (p = 0.315), τ^2 = 0.0008, I^2 = 15.05%			

**B) CV Mortality**			
	Placebo	Tirzepatide	GLP-1RA
Placebo	Placebo	1.33 (1.13; 1.56)	1.18 (1.09; 1.28)
Tirzepatide	0.75 (0.64; 0.88)	Tirzepatide	
GLP-1RA	0.85 (0.78; 0.91)		GLP-1RA
Q = 6.04 (p = 0.419), τ^2 = 0.0000, I^2 = 0.62%			

**C) All-Cause Mortality**			
	Placebo	Tirzepatide	GLP-1RA
Placebo	Placebo	1.37 (1.20; 1.55)	1.15 (1.08; 1.22)
Tirzepatide	0.73 (0.64; 0.83)	Tirzepatide	
GLP-1RA	0.87 (0.82; 0.92)		GLP-1RA
Q = 3.76 (p = 0.709), τ^2 = 0.0000, I^2 = 0%			

**D) Non-Fatal MI**			
	Placebo	Tirzepatide	GLP-1RA
Placebo	Placebo	1.28 (1.07; 1.51)	1.10 (1.01; 1.19)
Tirzepatide	0.78 (0.66; 0.93)	Tirzepatide	
GLP-1RA	0.91 (0.84; 0.99)		GLP-1RA
Q = 3.81 (p = 0.578), τ^2 = 0.0000, I^2 = 0%			

**E) Non-Fatal Stroke**			
	Placebo	Tirzepatide	GLP-1RA
Placebo	Placebo	1.30 (1.05; 1.60)	1.18 (1.06; 1.32)
Tirzepatide	0.77 (0.62; 0.95)	Tirzepatide	
GLP-1RA	0.85 (0.76; 0.94)		GLP-1RA
Q = 6.7 (p = 0.244), τ^2 = 0.0000, I^2 = 25.36%			

Supplemental Figure S3. Class-level network meta-analysis comparing tirzepatide, injectable GLP-1RA, and placebo following exclusion of short-acting and oral GLP-1RA trials (ELIXA, PIONEER 6, and SOUL). Outcomes shown include: (A) major adverse cardiovascular events (MACE), (B) cardiovascular (CV) mortality, (C) all-cause mortality, (D) non-fatal myocardial infarction (MI), and (E) non-fatal stroke. Hazard ratios (HRs) with 95% confidence intervals represent the row treatment versus the column treatment. Green shading denotes statistically significant benefit, red shading denotes statistically significant relative harm. Values less than 1 favor the active treatment, while values greater than 1 favor the comparator. Measures of between-study heterogeneity (Q statistic, τ^2, and I^2) are reported for each outcome.

**A) MACE**			
	Placebo	Tirzepatide	GLP-1RA
Placebo	Placebo	1.27 (1.14; 1.41)	1.17 (1.11; 1.22)
Tirzepatide	0.79 (0.71; 0.88)	Tirzepatide	
GLP-1RA	0.86 (0.82; 0.90)		GLP-1RA
Q = 7.09 (p = 0.42), τ^2 = 0.0008, I^2 = 1.25%			

**B) CV Mortality**			
	Placebo	Tirzepatide	GLP-1RA
Placebo	Placebo	1.29 (1.10; 1.51)	1.15 (1.07; 1.23)
Tirzepatide	0.78 (0.66; 0.91)	Tirzepatide	
GLP-1RA	0.87 (0.81; 0.94)		GLP-1RA
Q = 7.78 (p = 0.352), τ^2 = 0.0000, I^2 = 10.02%			

**C) All-Cause Mortality**			
	Placebo	Tirzepatide	GLP-1RA
Placebo	Placebo	1.35 (1.19; 1.53)	1.14 (1.07; 1.20)
Tirzepatide	0.74 (0.65; 0.84)	Tirzepatide	
GLP-1RA	0.88 (0.83; 0.93)		GLP-1RA
Q = 7.63 (p = 0.366), τ^2 = 0.0000, I^2 = 8.3%			

**D) Non-Fatal MI**			
	Placebo	Tirzepatide	GLP-1RA
Placebo	Placebo	1.32 (1.04; 1.66)	1.13 (1.03; 1.25)
Tirzepatide	0.76 (0.60; 0.96)	Tirzepatide	
GLP-1RA	0.88 (0.80; 0.98)		GLP-1RA
Q = 8.82 (p = 0.184), τ^2 = 0.0056, I^2 = 31.94%			

**E) Non-Fatal Stroke**			
	Placebo	Tirzepatide	GLP-1RA
Placebo	Placebo	1.33 (1.08; 1.63)	1.21 (1.09; 1.34)
Tirzepatide	0.75 (0.61; 0.93)	Tirzepatide	
GLP-1RA	0.83 (0.75; 0.92)		GLP-1RA
Q = 3.04 (p = 0.803), τ^2 = 0.0000, I^2 = 0%			

Supplemental Figure S4. Class-level network meta-analysis comparing tirzepatide, GLP-1 receptor agonists (GLP-1RA), and placebo after exclusion of trials with distinct study populations relative to SURPASS-CVOT, specifically ELIXA, which enrolled patients with recent acute coronary syndrome, and FLOW, a dedicated chronic kidney disease outcomes trial. Outcomes shown include: (A) major adverse cardiovascular events (MACE), (B) cardiovascular (CV) mortality, (C) all-cause mortality, (D) non-fatal myocardial infarction (MI), and (E) non-fatal stroke. Hazard ratios (HRs) with 95% confidence intervals represent the row treatment versus the column treatment. Green shading denotes statistically significant benefit, red shading denotes statistically significant relative harm. Values less than 1 favor the active treatment, while

values greater than 1 favor the comparator. Measures of between-study heterogeneity (Q statistic, τ^2, and I^2) are reported for each outcome.

Leave One Out Analysis – Class Level Analysis

Major Adverse Cardiovascular Events

Study Left Out	Tau-squared (τ^2)	I-squared (I^2)	Cochrane Q	Q p-value
**AMPLITUDE-O**	0.001	26.18	10.84	0.21
**ELIXA**	1.564	0	7.29	0.51
**EXSCEL**	0.003	33	11.94	0.15
**FLOW**	0.002	37.02	12.70	0.12
**Harmony Outcomes**	0.000	24.42	10.58	0.23
**LEADER**	0.004	39	13.12	0.11
**PIONEER 6**	0.002	37.43	12.79	0.12
**REWIND**	0.004	38.73	13.06	0.11
**SOUL**	0.004	38.81	13.07	0.11
**SURPASS CVOT**	0.002	31.38	13.12	0.16
**SUSTAIN 6**	0.001	30.01	11.43	0.18

Supplemental Table S5. Leave-one-out sensitivity analysis at the class-level for major adverse cardiovascular events (MACE) showing changes in between-study heterogeneity (τ^2 and I^2) following sequential exclusion of individual trials from the tirzepatide versus placebo comparison.

Study Left Out	Comparison	HR	Lower 95% CI	Upper 95% CI	Base HR	Delta HR
**AMPLITUDE-O**	Tirzepatide vs Placebo	0.80	0.71	0.91	0.79	0.008
**ELIXA**	Tirzepatide vs Placebo	0.79	0.71	0.88	0.79	-0.008
**EXSCEL**	Tirzepatide vs Placebo	0.78	0.67	0.92	0.79	-0.011
**FLOW**	Tirzepatide vs Placebo	0.80	0.69	0.92	0.79	0.002
**Harmony Outcomes**	Tirzepatide vs Placebo	0.81	0.73	0.90	0.79	0.014
**LEADER**	Tirzepatide vs Placebo	0.79	0.67	0.93	0.79	-0.005
**PIONEER 6**	Tirzepatide vs Placebo	0.80	0.69	0.92	0.79	0.002
**REWIND**	Tirzepatide vs Placebo	0.79	0.67	0.93	0.79	-0.006
**SOUL**	Tirzepatide vs Placebo	0.79	0.67	0.94	0.79	-0.003

**SURPASS CVOT**	Tirzepatide vs Placebo	NA	NA	NA	0.79	NA
**SUSTAIN 6**	Tirzepatide vs Placebo	0.80	0.70	0.92	0.79	0.006

Supplemental Table S6. Leave-one-out sensitivity analysis at the class-level for major adverse cardiovascular events (MACE) comparing tirzepatide versus placebo, presenting hazard ratios with 95% confidence intervals after sequential removal of each individual trial.

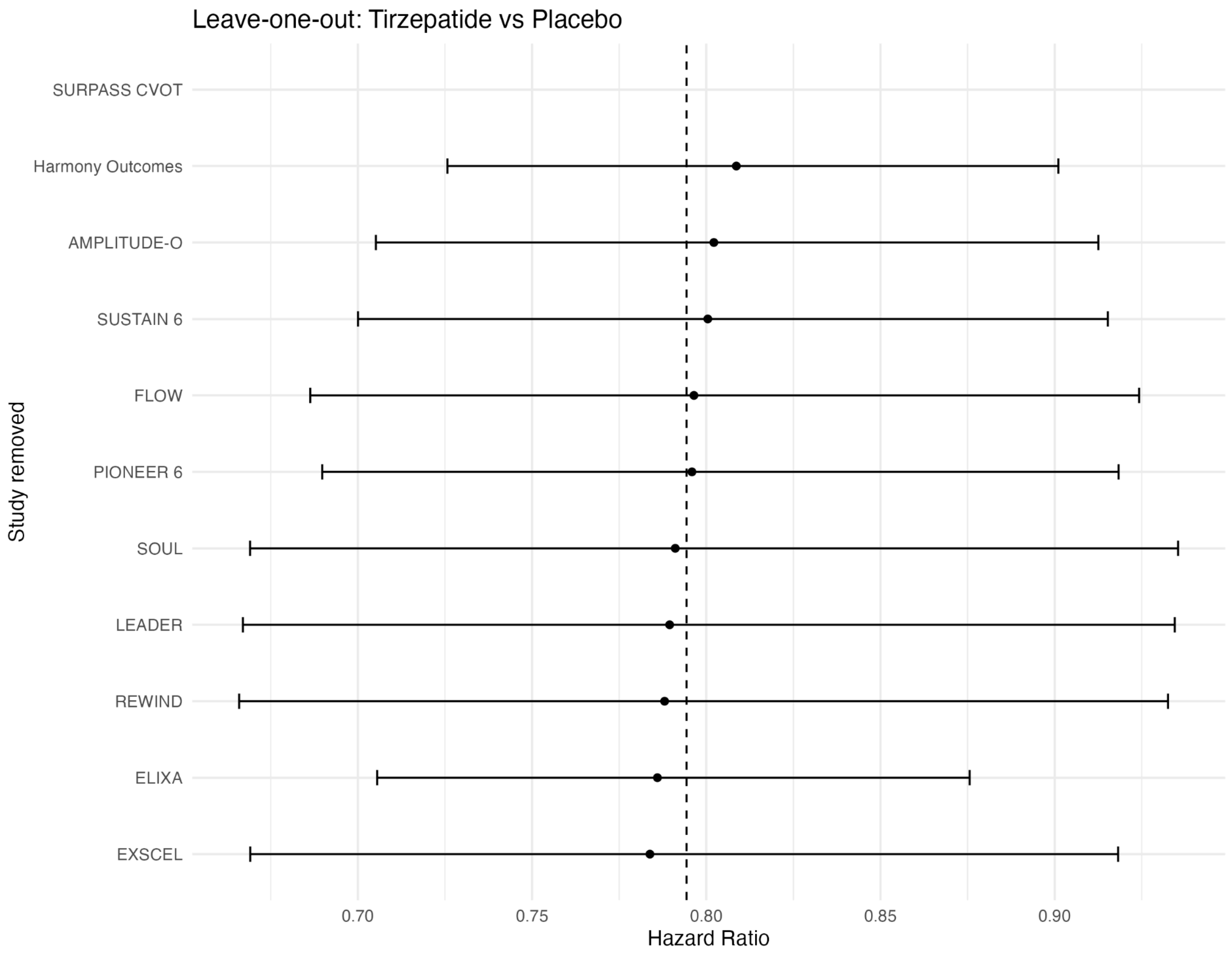

Supplemental Figure S5. Leave-one-out sensitivity analysis at the class-level for major adverse cardiovascular events (MACE) comparing tirzepatide versus placebo, demonstrating stable effect estimates across exclusion of individual trials.

Cardiovascular Mortality

**Study Left Out**	**Tau-squared (τ²)**	**I-squared (I²)**	**Cochrane Q**	**Q p-value**
**AMPLITUDE-O**	0.00	25.46	10.73	0.22

**ELIXA**	0.001	23.97	10.52	0.23
**EXSCEL**	0.003	31.65	11.70	0.16
**FLOW**	0.000	8.17	8.71	0.37
**Harmony Outcomes**	0.001	29.87	11.41	0.18
**LEADER**	0.000	20.86	10.11	0.26
**PIONEER 6**	0.000	3.51	8.29	0.41
**REWIND**	0.002	28.99	11.27	0.19
**SOUL**	0.001	25.65	10.76	0.22
**SURPASS CVOT**	0.000	23.50	11.76	0.23
**SUSTAIN 6**	0.0005	29.86	11.41	0.18

Supplemental Table S7. Leave-one-out sensitivity analysis at the class-level for cardiovascular mortality showing changes in between-study heterogeneity (τ^2 and I^2) following sequential exclusion of individual trials from the tirzepatide versus placebo comparison.

**Study Left Out**	**Comparison**	**HR**	**Lower 95% CI**	**Upper 95% CI**	**Base HR**	**Delta HR**
**AMPLITUDE-O**	Tirzepatide vs Placebo	0.78	0.66	0.91	0.77	0.005
**ELIXA**	Tirzepatide vs Placebo	0.76	0.65	0.90	0.77	-0.009
**EXSCEL**	Tirzepatide vs Placebo	0.76	0.63	0.93	0.77	-0.007
**FLOW**	Tirzepatide vs Placebo	0.78	0.67	0.92	0.77	0.013
**Harmony Outcomes**	Tirzepatide vs Placebo	0.76	0.64	0.91	0.77	-0.006
**LEADER**	Tirzepatide vs Placebo	0.78	0.67	0.92	0.77	0.014
**PIONEER 6**	Tirzepatide vs Placebo	0.78	0.66	0.91	0.77	0.005
**REWIND**	Tirzepatide vs Placebo	0.76	0.63	0.91	0.77	-0.011
**SOUL**	Tirzepatide vs Placebo	0.76	0.64	0.90	0.77	-0.013
**SURPASS CVOT**	Tirzepatide vs Placebo	NA	NA	NA	0.77	NA
**SUSTAIN 6**	Tirzepatide vs Placebo	0.77	0.65	0.90	0.77	-0.003

Supplemental Table S8. Leave-one-out sensitivity analysis at the class-level for cardiovascular mortality comparing tirzepatide versus placebo, presenting hazard ratios with 95% confidence intervals after sequential removal of each individual trial.

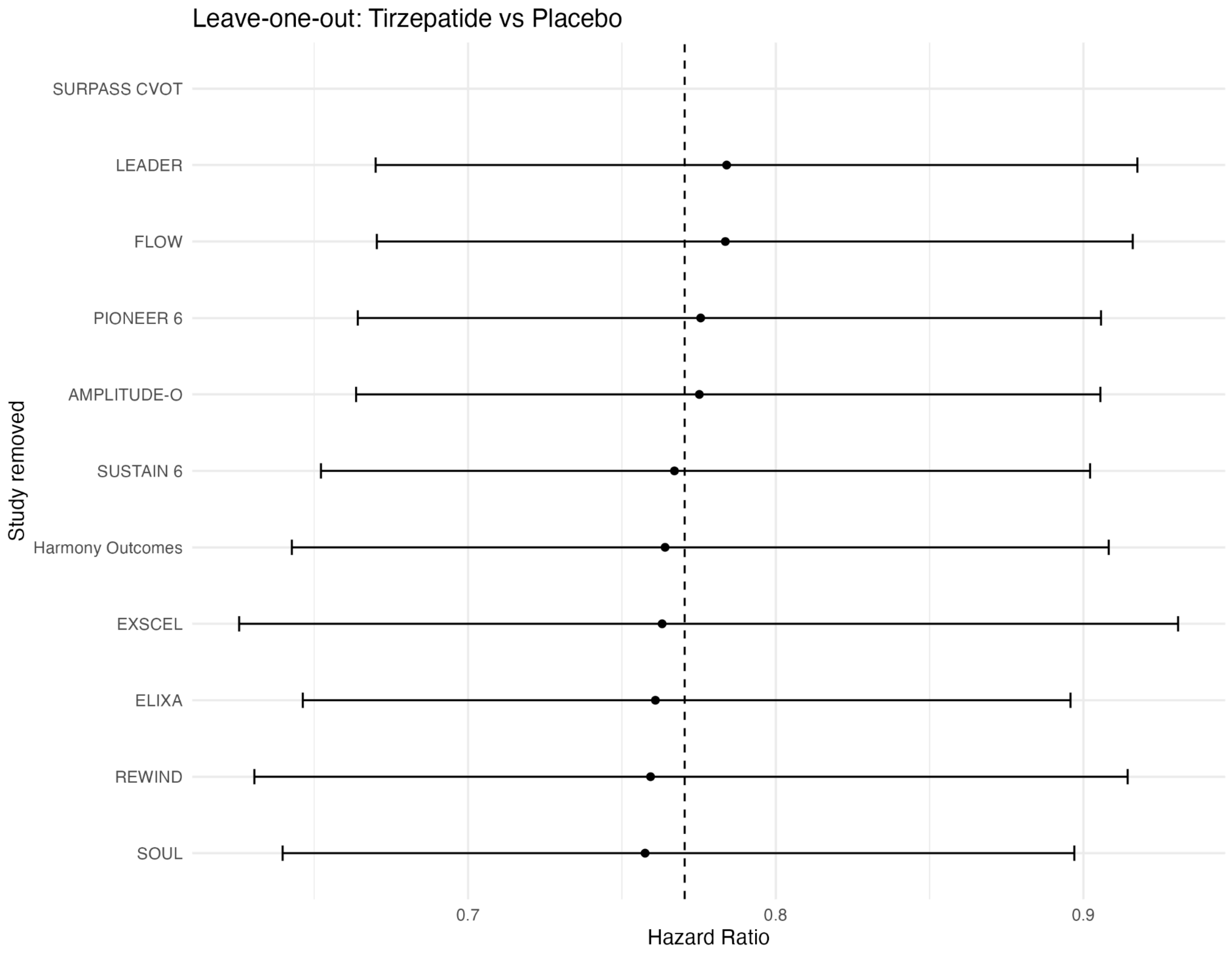

Supplemental Figure S6. Leave-one-out sensitivity analysis at the class-level for cardiovascular mortality comparing tirzepatide versus placebo, demonstrating stable effect estimates across exclusion of individual trials.

All-Cause Mortality

Study Left Out	Tau-squared (τ²)	I-squared (I²)	Cochrane Q	Q p-value
**AMPLITUDE-O**	0.000	7.30	8.63	0.37
**ELIXA**	0.000	7.89	8.69	0.37
**EXSCEL**	0.000	12.16	9.11	0.33
**FLOW**	0.000	0.89	8.07	0.43
**Harmony Outcomes**	0.000	6.63	8.57	0.38
**LEADER**	0.000	11.15	9.00	0.34
**PIONEER 6**	0.000	0	4.65	0.79
**REWIND**	0.000	11.45	9.03	0.34

**SOUL**	0.000	9.51	8.84	0.36
**SURPASS CVOT**	0.000	2.76	9.26	0.41
**SUSTAIN 6**	0.000	2.97	8.24	0.41

Supplemental Table S9. Leave-one-out sensitivity analysis at the class-level for all-cause mortality showing changes in between-study heterogeneity (τ^2 and I^2) following sequential exclusion of individual trials from the tirzepatide versus placebo comparison.

**Study Left Out**	**Comparison**	**HR**	**Lower 95% CI**	**Upper 95% CI**	**Base HR**	**Delta HR**
**AMPLITUDE-O**	Tirzepatide vs Placebo	0.74	0.65	0.84	0.74	0.003
**ELIXA**	Tirzepatide vs Placebo	0.73	0.65	0.83	0.74	-0.004
**EXSCEL**	Tirzepatide vs Placebo	0.74	0.65	0.84	0.74	0.004
**FLOW**	Tirzepatide vs Placebo	0.74	0.67	0.84	0.74	0.006
**Harmony Outcomes**	Tirzepatide vs Placebo	0.73	0.65	0.83	0.74	-0.004
**LEADER**	Tirzepatide vs Placebo	0.74	0.65	0.84	0.74	0.004
**PIONEER 6**	Tirzepatide vs Placebo	0.74	0.66	0.84	0.74	0.004
**REWIND**	Tirzepatide vs Placebo	0.73	0.65	0.83	0.74	-0.004
**SOUL**	Tirzepatide vs Placebo	0.73	0.64	0.83	0.74	-0.006
**SURPASS CVOT**	Tirzepatide vs Placebo	NA	NA	NA	0.74	NA
**SUSTAIN 6**	Tirzepatide vs Placebo	0.73	0.65	0.83	0.74	-0.003

Supplemental Table S10. Leave-one-out sensitivity analysis at the class-level for all-cause mortality events comparing tirzepatide versus placebo, presenting hazard ratios with 95% confidence intervals after sequential removal of each individual trial.

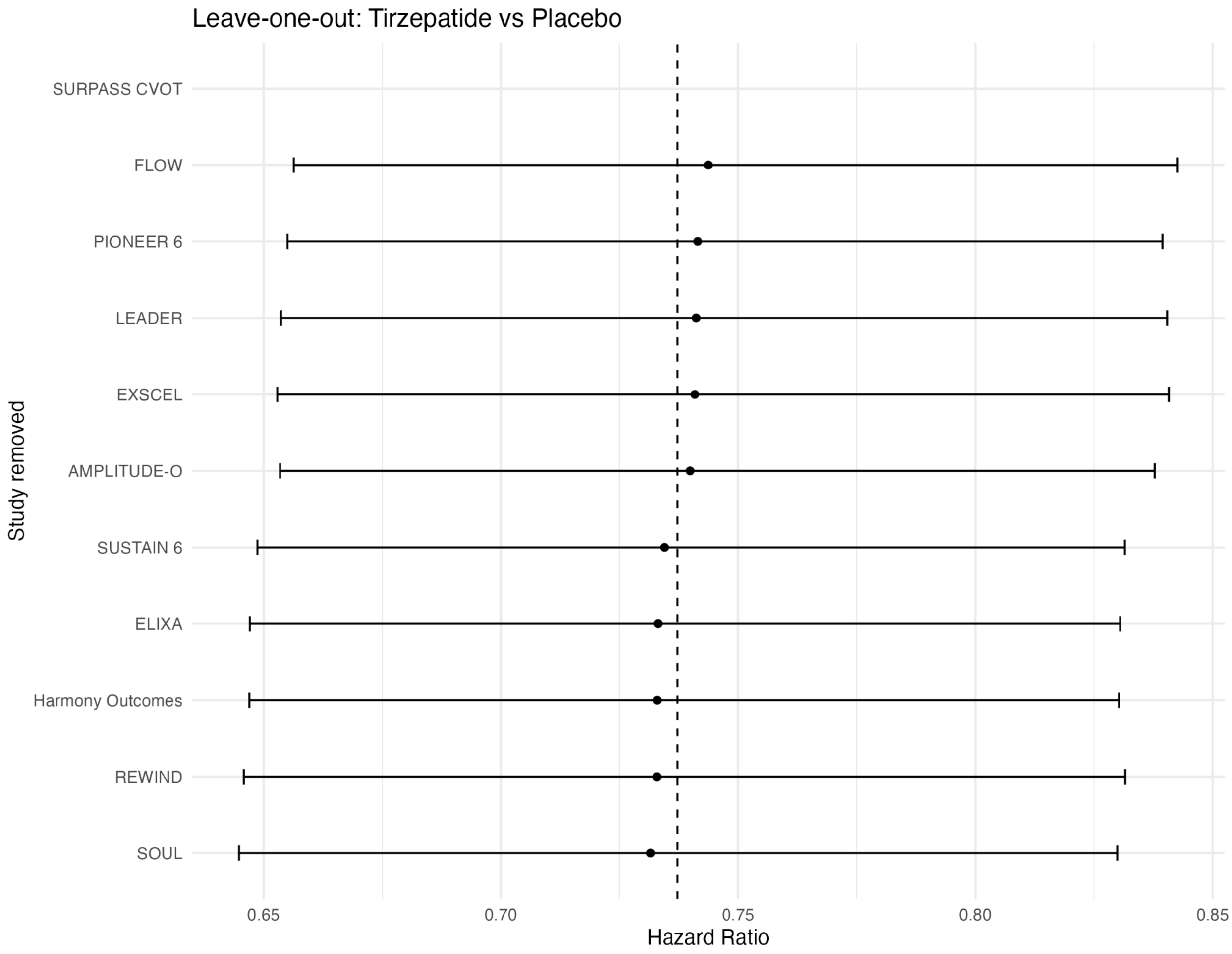

Supplemental Figure S7. Leave-one-out sensitivity analysis at the class-level for all-cause mortality comparing tirzepatide versus placebo, demonstrating stable effect estimates across exclusion of individual trials.

Non-Fatal Myocardial Infarction

**Study Left Out**	**Tau-squared (τ²)**	**I-squared (I²)**	**Cochrane Q**	**Q p-value**
**AMPLITUDE-O**	0.006	35.32	10.82	0.15
**ELIXA**	0.005	23.40	9.14	0.24
**EXSCEL**	0.008	31.99	10.29	0.17
**FLOW**	0.006	37.08	11.13	0.13
**LEADER**	0.009	38.61	11.40	0.12
**PIONEER 6**	0.006	33.01	10.45	0.16
**REWIND**	0.008	37.86	11.26	0.13
**SOUL**	0.000	0.00	6.34	0.50

**SURPASS CVOT**	0.006	31.09	11.61	0.17
**SUSTAIN 6**	0.006	32.73	10.41	0.17

Supplemental Table S11. Leave-one-out sensitivity analysis at the class-level for non-fatal myocardial infarction showing changes in between-study heterogeneity (τ^2 and I^2) following sequential exclusion of individual trials from the tirzepatide versus placebo comparison.

Study Left Out	Comparison	HR	Lower 95% CI	Upper 95% CI	Base HR	Delta HR
**AMPLITUDE-O**	Tirzepatide vs Placebo	0.78	0.62	0.99	0.77	0.007
**ELIXA**	Tirzepatide vs Placebo	0.76	0.60	0.95	0.77	-0.019
**EXSCEL**	Tirzepatide vs Placebo	0.76	0.59	0.97	0.77	-0.016
**FLOW**	Tirzepatide vs Placebo	0.78	0.61	0.99	0.77	0.005
**LEADER**	Tirzepatide vs Placebo	0.77	0.59	1.01	0.77	0.00
**PIONEER 6**	Tirzepatide vs Placebo	0.77	0.61	0.97	0.77	-0.008
**REWIND**	Tirzepatide vs Placebo	0.76	0.59	0.99	0.77	-0.010
**SOUL**	Tirzepatide vs Placebo	0.81	0.68	0.95	0.77	0.033
**SURPASS CVOT**	Tirzepatide vs Placebo	NA	NA	NA	0.77	NA
**SUSTAIN 6**	Tirzepatide vs Placebo	0.78	0.62	0.98	0.77	0.008

Supplemental Table S12. Leave-one-out sensitivity analysis at the class-level for non-fatal myocardial infarction events comparing tirzepatide versus placebo, presenting hazard ratios with 95% confidence intervals after sequential removal of each individual trial.

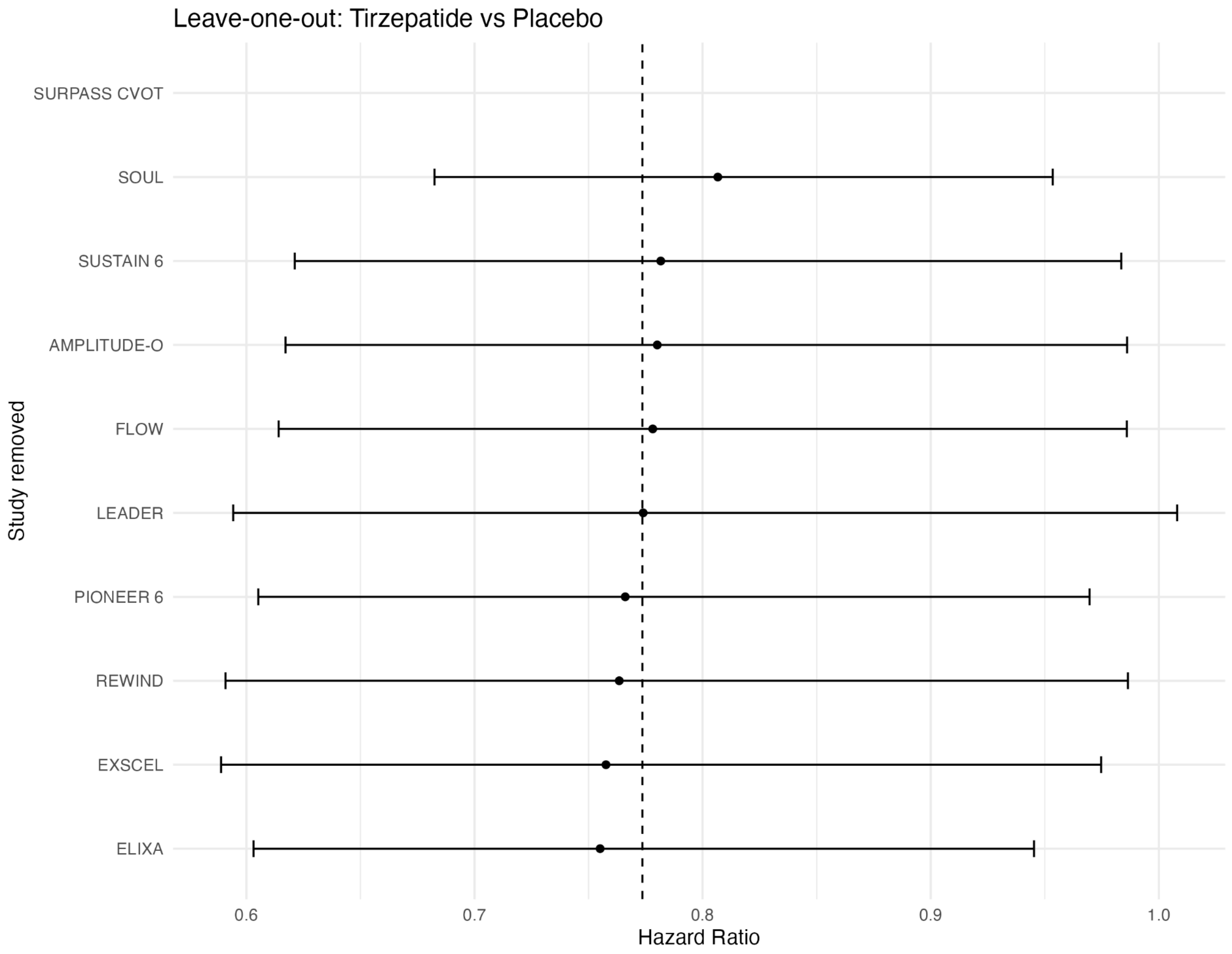

Supplemental Figure S8. Leave-one-out sensitivity analysis at the class-level for non-fatal myocardial infarction comparing tirzepatide versus placebo, demonstrating stable effect estimates across exclusion of individual trials.

Non-Fatal Stroke

**Study Left Out**	**Tau-squared (τ^2)**	**I-squared (I^2)**	**Cochrane Q**	**Q p-value**
**AMPLITUDE-O**	0.000	22.89	9.08	0.25
**ELIXA**	0.000	0.00	6.92	0.44
**EXSCEL**	0.005	23.28	9.12	0.24
**FLOW**	0.000	0.00	5.74	0.57
**LEADER**	0.003	23.18	9.11	0.24
**PIONEER 6**	0.000	22.26	9.00	0.25
**REWIND**	0.000	6.80	7.51	0.38
**SOUL**	0.003	23.59	9.16	0.24

**SURPASS CVOT**	0.000	12.85	9.18	0.33
**SUSTAIN 6**	0.000	0.00	6.97	0.43

Supplemental Table S13. Leave-one-out sensitivity analysis at the class-level for non-fatal stroke showing changes in between-study heterogeneity (τ^2 and I^2) following sequential exclusion of individual trials from the tirzepatide versus placebo comparison.

Study Left Out	Comparison	HR	Lower 95% CI	Upper 95% CI	Base HR	Delta HR
**AMPLITUDE-O**	Tirzepatide vs Placebo	0.79	0.65	0.97	0.79	0.002
**ELIXA**	Tirzepatide vs Placebo	0.77	0.63	0.95	0.79	-0.016
**EXSCEL**	Tirzepatide vs Placebo	0.80	0.62	1.03	0.79	0.007
**FLOW**	Tirzepatide vs Placebo	0.77	0.63	0.95	0.79	-0.018
**LEADER**	Tirzepatide vs Placebo	0.79	0.62	0.99	0.79	-0.003
**PIONEER 6**	Tirzepatide vs Placebo	0.79	0.65	0.97	0.79	0.002
**REWIND**	Tirzepatide vs Placebo	0.81	0.66	1.00	0.79	0.023
**SOUL**	Tirzepatide vs Placebo	0.79	0.62	0.99	0.79	-0.000
**SURPASS CVOT**	Tirzepatide vs Placebo	NA	NA	NA	0.79	NA
**SUSTAIN 6**	Tirzepatide vs Placebo	0.80	0.65	0.98	0.79	0.011

Supplemental Table S14. Leave-one-out sensitivity analysis at the class-level for non-fatal stroke events comparing tirzepatide versus placebo, presenting hazard ratios with 95% confidence intervals after sequential removal of each individual trial.

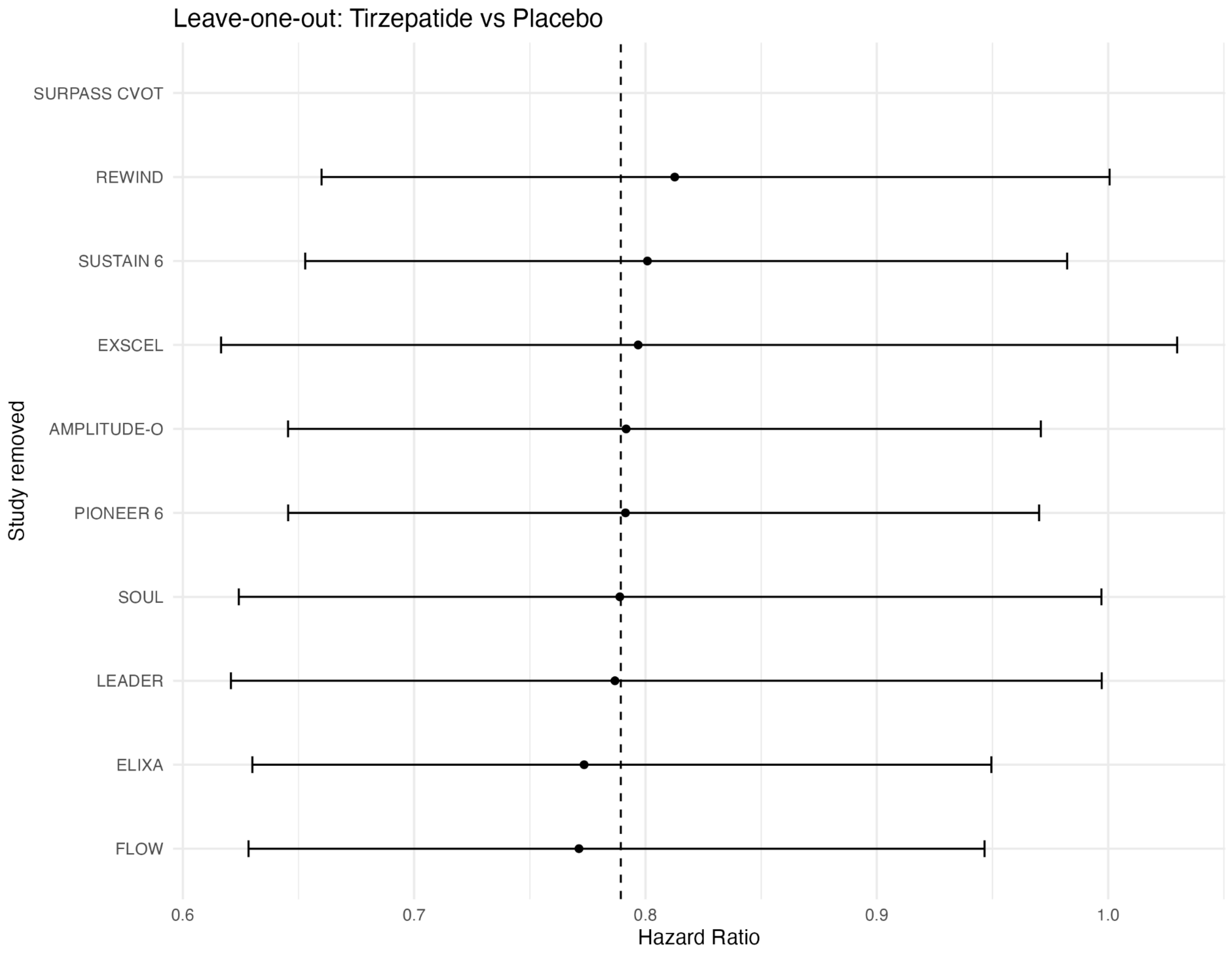

Supplemental Figure S9. Leave-one-out sensitivity analysis at the class-level for non-fatal stroke comparing tirzepatide versus placebo, demonstrating stable effect estimates across exclusion of individual trials.

Leave One Out Analysis – Agent Level Analysis

Major Adverse Cardiovascular Events

Study Left Out	Tau-squared (τ^2)	I-squared (I^2)	Cochrane Q	Q p-value
**AMPLITUDE-O**	0.000	0.00	1.33	0.72
**ELIXA**	0.000	0.00	1.33	0.72
**EXSCEL**	0.000	0.00	1.33	0.72
**FLOW**	0.000	0.00	1.30	0.52
**Harmony Outcomes**	0.000	0.00	1.33	0.72
**LEADER**	0.000	0.00	1.33	0.72
**PIONEER 6**	0.000	0.00	1.23	0.54

**REWIND**	NA	0.00	NA	NA
**SOUL**	0.000	0.00	0.43	0.81
**SURPASS CVOT**	0.000	0.00	1.33	0.72
**SUSTAIN 6**	0.000	0.00	0.36	0.83

Supplemental Table S15. Leave-one-out sensitivity analysis at the agent-level for major adverse cardiovascular events (MACE) showing changes in between-study heterogeneity (τ^2 and I^2) following sequential exclusion of individual trials from the tirzepatide versus placebo comparison.

Study Left Out	Comparison	HR	Lower 95% CI	Upper 95% CI	Base HR	Delta HR
**AMPLITUDE-O**	Tirzepatide vs Placebo	0.81	0.70	0.94	0.81	0.000
**AMPLITUDE-O**	Tirzepatide vs Dulaglutide	0.92	0.83	1.01	0.92	0.000
**AMPLITUDE-O**	Tirzepatide vs Albiglutide	1.04	0.85	1.27	1.042	0.000
**AMPLITUDE-O**	Tirzepatide vs Efpeglenatide	NA	NA	NA	1.11	NA
**AMPLITUDE-O**	Tirzepatide vs Exenatide	0.89	0.75	1.06	0.89	0.000
**AMPLITUDE-O**	Tirzepatide vs Liraglutide	0.93	0.78	1.12	0.93	0.000
**AMPLITUDE-O**	Tirzepatide vs Lixisenatide	0.79	0.65	0.97	0.79	0.000
**AMPLITUDE-O**	Tirzepatide vs Semaglutide	0.97	0.82	1.15	0.97	0.000
**ELIXA**	Tirzepatide vs Placebo	0.81	0.70	0.94	0.81	0.000
**ELIXA**	Tirzepatide vs Dulaglutide	0.92	0.83	1.01	0.92	0.000
**ELIXA**	Tirzepatide vs Albiglutide	1.04	0.85	1.27	1.04	0.000
**ELIXA**	Tirzepatide vs Efpeglenatide	1.11	0.84	1.46	1.11	0.000
**ELIXA**	Tirzepatide vs Exenatide	0.89	0.75	1.06	0.89	0.000
**ELIXA**	Tirzepatide vs Liraglutide	0.93	0.78	1.12	0.93	0.000
**ELIXA**	Tirzepatide vs Lixisenatide	NA	NA	NA	0.79	NA
**ELIXA**	Tirzepatide vs Semaglutide	0.97	0.82	1.15	0.97	0.000
**EXSCEL**	Tirzepatide vs Placebo	0.81	0.70	0.94	0.81	0.000
**EXSCEL**	Tirzepatide vs Dulaglutide	0.92	0.83	1.01	0.92	0.000
**EXSCEL**	Tirzepatide vs Albiglutide	1.04	0.85	1.27	1.04	0.000

**EXSCEL**	Tirzepatide vs Efpeglenatide	1.11	0.84	1.46	1.11	0.000
**EXSCEL**	Tirzepatide vs Exenatide	NA	NA	NA	0.89	NA
**EXSCEL**	Tirzepatide vs Liraglutide	0.93	0.78	1.12	0.93	0.000
**EXSCEL**	Tirzepatide vs Lixisenatide	0.79	0.65	0.97	0.79	0.000
**EXSCEL**	Tirzepatide vs Semaglutide	0.97	0.82	1.15	0.97	0.000
**FLOW**	Tirzepatide vs Placebo	0.81	0.70	0.94	0.81	0.000
**FLOW**	Tirzepatide vs Dulaglutide	0.92	0.83	1.01	0.92	0.000
**FLOW**	Tirzepatide vs Albiglutide	1.04	0.85	1.27	1.04	0.000
**FLOW**	Tirzepatide vs Efpeglenatide	1.11	0.84	1.46	1.11	0.000
**FLOW**	Tirzepatide vs Exenatide	0.89	0.75	1.06	0.89	0.000
**FLOW**	Tirzepatide vs Liraglutide	0.93	0.78	1.12	0.93	0.000
**FLOW**	Tirzepatide vs Lixisenatide	0.79	0.65	0.97	0.79	0.000
**FLOW**	Tirzepatide vs Semaglutide	0.97	0.81	1.16	0.97	-0.004
**Harmony Outcomes**	Tirzepatide vs Placebo	0.81	0.70	0.94	0.81	0.000
**Harmony Outcomes**	Tirzepatide vs Dulaglutide	0.92	0.83	1.01	0.92	0.000
**Harmony Outcomes**	Tirzepatide vs Albiglutide	NA	NA	NA	1.04	NA
**Harmony Outcomes**	Tirzepatide vs Efpeglenatide	1.11	0.84	1.46	1.11	0.000
**Harmony Outcomes**	Tirzepatide vs Exenatide	0.89	0.75	1.06	0.89	0.000
**Harmony Outcomes**	Tirzepatide vs Liraglutide	0.93	0.78	1.12	0.93	0.000
**Harmony Outcomes**	Tirzepatide vs Lixisenatide	0.79	0.65	0.97	0.79	0.000
**Harmony Outcomes**	Tirzepatide vs Semaglutide	0.97	0.82	1.15	0.97	0.000
**LEADER**	Tirzepatide vs Placebo	0.81	0.70	0.94	0.81	0.000
**LEADER**	Tirzepatide vs Dulaglutide	0.92	0.83	1.01	0.92	0.000
**LEADER**	Tirzepatide vs Albiglutide	1.04	0.85	1.27	1.04	0.000
**LEADER**	Tirzepatide vs Efpeglenatide	1.11	0.84	1.46	1.11	0.000
**LEADER**	Tirzepatide vs Exenatide	0.89	0.75	1.06	0.89	0.000

**LEADER**	Tirzepatide vs Liraglutide	NA	NA	NA	0.93	NA
**LEADER**	Tirzepatide vs Lixisenatide	0.79	0.65	0.97	0.79	0.000
**LEADER**	Tirzepatide vs Semaglutide	0.97	0.82	1.15	0.97	0.000
**PIONEER 6**	Tirzepatide vs Placebo	0.81	0.70	0.94	0.81	0.000
**PIONEER 6**	Tirzepatide vs Dulaglutide	0.92	0.83	1.01	0.92	0.000
**PIONEER 6**	Tirzepatide vs Albiglutide	1.04	0.85	1.27	1.04	0.000
**PIONEER 6**	Tirzepatide vs Efpeglenatide	1.11	0.84	1.46	1.11	0.000
**PIONEER 6**	Tirzepatide vs Exenatide	0.89	0.748349873624205	1.05767813076854	0.89	0.000
**PIONEER 6**	Tirzepatide vs Liraglutide	0.93	0.775729083814131	1.11632954581779	0.93	0.000
**PIONEER 6**	Tirzepatide vs Lixisenatide	0.79	0.649932209437207	0.969332100547122	0.79	0.000
**PIONEER 6**	Tirzepatide vs Semaglutide	0.97	0.8183614759762	1.15055015106232	0.97	-0.004
**REWIND**	Tirzepatide vs Placebo	NA	NA	NA	0.81	NA
**REWIND**	Tirzepatide vs Dulaglutide	NA	NA	NA	0.92	NA
**REWIND**	Tirzepatide vs Albiglutide	NA	NA	NA	1.04	NA
**REWIND**	Tirzepatide vs Efpeglenatide	NA	NA	NA	1.11	NA
**REWIND**	Tirzepatide vs Exenatide	NA	NA	NA	0.89	NA
**REWIND**	Tirzepatide vs Liraglutide	NA	NA	NA	0.93	NA
**REWIND**	Tirzepatide vs Lixisenatide	NA	NA	NA	0.79	NA
**REWIND**	Tirzepatide vs Semaglutide	NA	NA	NA	0.97	NA
**SOUL**	Tirzepatide vs Placebo	0.81	0.699797520437937	0.936631155237327	0.81	0.000
**SOUL**	Tirzepatide vs Dulaglutide	0.92	0.834001765078154	1.01486595765259	0.92	0.000
**SOUL**	Tirzepatide vs Albiglutide	1.04	0.847933902826999	1.27054424584225	1.04	0.000
**SOUL**	Tirzepatide vs Efpeglenatide	1.11	0.844204073773008	1.456960693019	1.11	0.000
**SOUL**	Tirzepatide vs Exenatide	0.89	0.748349873617016	1.0576781307787	0.89	0.000
**SOUL**	Tirzepatide vs Liraglutide	0.93	0.775729083807045	1.11632954582796	0.93	0.000
**SOUL**	Tirzepatide vs Lixisenatide	0.79	0.649932209431808	0.96933210055518	0.80	0.000

**SOUL**	Tirzepatide vs Semaglutide	1.02	0.840059194334327	1.24830247743021	0.97	0.050
**SURPASS CVOT**	Tirzepatide vs Placebo	NA	NA	NA	0.81	NA
**SURPASS CVOT**	Tirzepatide vs Dulaglutide	NA	NA	NA	0.92	NA
**SURPASS CVOT**	Tirzepatide vs Albiglutide	NA	NA	NA	1.04	NA
**SURPASS CVOT**	Tirzepatide vs Efpeglenatide	NA	NA	NA	1.11	NA
**SURPASS CVOT**	Tirzepatide vs Exenatide	NA	NA	NA	0.89	NA
**SURPASS CVOT**	Tirzepatide vs Liraglutide	NA	NA	NA	0.93	NA
**SURPASS CVOT**	Tirzepatide vs Lixisenatide	NA	NA	NA	0.79	NA
**SURPASS CVOT**	Tirzepatide vs Semaglutide	NA	NA	NA	0.97	NA
**SUSTAIN 6**	Tirzepatide vs Placebo	0.81	0.70	0.94	0.81	0.000
**SUSTAIN 6**	Tirzepatide vs Dulaglutide	0.92	0.83	1.01	0.92	0.000
**SUSTAIN 6**	Tirzepatide vs Albiglutide	1.04	0.85	1.27	1.04	0.000
**SUSTAIN 6**	Tirzepatide vs Efpeglenatide	1.11	0.84	1.46	1.11	0.000
**SUSTAIN 6**	Tirzepatide vs Exenatide	0.89	0.75	1.06	0.89	0.000
**SUSTAIN 6**	Tirzepatide vs Liraglutide	0.93	0.78	1.12	0.93	0.000
**SUSTAIN 6**	Tirzepatide vs Lixisenatide	0.79	0.65	0.97	0.79	0.000
**SUSTAIN 6**	Tirzepatide vs Semaglutide	0.96	0.81	1.14	0.97	-0.015

Supplemental Table S16. Leave-one-out sensitivity analysis at the agent-level for major adverse cardiovascular events (MACE) comparing tirzepatide versus placebo, presenting hazard ratios with 95% confidence intervals after sequential removal of each individual trial.

Cardiovascular Mortality

**Study Left Out**	**Tau-squared (τ²)**	**I-squared (I²)**	**Cochrane Q**	**Q p-value**

**AMPLITUDE-O**	0.026	58.85	7.29	0.06
**ELIXA**	0.026	58.85	7.29	0.06
**EXSCEL**	0.026	58.85	7.29	0.06
**FLOW**	0.036	52.97	4.25	0.12
**Harmony Outcomes**	0.026	58.85	7.29	0.06
**LEADER**	0.026	58.85	7.29	0.06
**PIONEER 6**	0.015	49.85	3.99	0.14
**REWIND**	NA	NA	NA	NA
**SOUL**	0.027	46.04	3.71	0.16
**SURPASS CVOT**	0.026	58.85	7.29	0.06
**SUSTAIN 6**	0.046	70.41	6.76	0.03

Supplemental Table S17. Leave-one-out sensitivity analysis at the agent-level for cardiovascular mortality showing changes in between-study heterogeneity (τ^2 and I^2) following sequential exclusion of individual trials from the tirzepatide versus placebo comparison.

Study Left Out	Comparison	HR	Lower 95% CI	Upper 95% CI	Base HR	Delta HR
**AMPLITUDE-O**	Tirzepatide vs Placebo	0.81	0.50	1.32	0.81	0.000
**AMPLITUDE-O**	Tirzepatide vs Dulaglutide	0.89	0.63	1.26	0.89	0.000
**AMPLITUDE-O**	Tirzepatide vs Albiglutide	0.87	0.46	1.64	0.87	0.000
**AMPLITUDE-O**	Tirzepatide vs Efpeglenatide	NA	NA	NA	1.12	NA
**AMPLITUDE-O**	Tirzepatide vs Exenatide	0.92	0.50	1.68	0.92	0.000
**AMPLITUDE-O**	Tirzepatide vs Liraglutide	1.04	0.56	1.91	1.04	0.000
**AMPLITUDE-O**	Tirzepatide vs Lixisenatide	0.83	0.44	1.55	0.83	0.000
**AMPLITUDE-O**	Tirzepatide vs Semaglutide	1.01	0.59	1.72	1.01	0.000
**ELIXA**	Tirzepatide vs Placebo	0.81	0.50	1.32	0.81	0.000
**ELIXA**	Tirzepatide vs Dulaglutide	0.89	0.63	1.26	0.89	0.000
**ELIXA**	Tirzepatide vs Albiglutide	0.87	0.46	1.64	0.87	0.000
**ELIXA**	Tirzepatide vs Efpeglenatide	1.12	0.57	2.24	1.12	0.000

**ELIXA**	Tirzepatide vs Exenatide	0.92	0.50	1.68	0.92	0.000
**ELIXA**	Tirzepatide vs Liraglutide	1.04	0.56	1.91	1.04	0.000
**ELIXA**	Tirzepatide vs Lixisenatide	NA	NA	NA	0.83	NA
**ELIXA**	Tirzepatide vs Semaglutide	1.01	0.59	1.72	1.01	0.000
**EXSCEL**	Tirzepatide vs Placebo	0.81	0.50	1.32	0.81	0.000
**EXSCEL**	Tirzepatide vs Dulaglutide	0.89	0.63	1.26	0.89	0.000
**EXSCEL**	Tirzepatide vs Albiglutide	0.87	0.46	1.64	0.87	0.000
**EXSCEL**	Tirzepatide vs Efpeglenatide	1.12	0.57	2.24	1.12	0.000
**EXSCEL**	Tirzepatide vs Exenatide	NA	NA	NA	0.92	NA
**EXSCEL**	Tirzepatide vs Liraglutide	1.04	0.56	1.91	1.04	0.000
**EXSCEL**	Tirzepatide vs Lixisenatide	0.83	0.44	1.55	0.83	0.000
**EXSCEL**	Tirzepatide vs Semaglutide	1.01	0.59	1.72	1.01	0.000
**FLOW**	Tirzepatide vs Placebo	0.81	0.46	1.43	0.81	0.000
**FLOW**	Tirzepatide vs Dulaglutide	0.89	0.60	1.33	0.89	0.000
**FLOW**	Tirzepatide vs Albiglutide	0.87	0.42	1.79	0.87	0.000
**FLOW**	Tirzepatide vs Efpeglenatide	1.12	0.52	2.43	1.12	0.000
**FLOW**	Tirzepatide vs Exenatide	0.92	0.46	1.84	0.92	0.000
**FLOW**	Tirzepatide vs Liraglutide	1.04	0.52	2.09	1.04	0.000
**FLOW**	Tirzepatide vs Lixisenatide	0.83	0.40	1.69	0.83	0.000
**FLOW**	Tirzepatide vs Semaglutide	0.96	0.51	1.82	1.01	-0.046
**Harmony Outcomes**	Tirzepatide vs Placebo	0.81	0.50	1.32	0.81	0.000

**Harmony Outcomes**	Tirzepatide vs Dulaglutide	0.89	0.63	1.26	0.89	0.000
**Harmony Outcomes**	Tirzepatide vs Albiglutide	NA	NA	NA	0.87	NA
**Harmony Outcomes**	Tirzepatide vs Efpeglenatide	1.12	0.57	2.24	1.12	0.000
**Harmony Outcomes**	Tirzepatide vs Exenatide	0.92	0.50	1.68	0.92	0.000
**Harmony Outcomes**	Tirzepatide vs Liraglutide	1.04	0.56	1.91	1.04	0.000
**Harmony Outcomes**	Tirzepatide vs Lixisenatide	0.83	0.44	1.55	0.83	0.000
**Harmony Outcomes**	Tirzepatide vs Semaglutide	1.01	0.59	1.72	1.01	0.000
**LEADER**	Tirzepatide vs Placebo	0.81	0.50	1.32	0.81	0.000
**LEADER**	Tirzepatide vs Dulaglutide	0.89	0.63	1.26	0.89	0.000
**LEADER**	Tirzepatide vs Albiglutide	0.87	0.46	1.64	0.87	0.000
**LEADER**	Tirzepatide vs Efpeglenatide	1.12	0.57	2.24	1.12	0.000
**LEADER**	Tirzepatide vs Exenatide	0.92	0.50	1.68	0.92	0.000
**LEADER**	Tirzepatide vs Liraglutide	NA	NA	NA	1.04	NA
**LEADER**	Tirzepatide vs Lixisenatide	0.83	0.44	1.55	0.83	0.000
**LEADER**	Tirzepatide vs Semaglutide	1.01	0.59	1.72	1.01	0.000
**PIONEER 6**	Tirzepatide vs Placebo	0.81	0.54	1.21	0.81	0.000
**PIONEER 6**	Tirzepatide vs Dulaglutide	0.89	0.67	1.18	0.89	0.000
**PIONEER 6**	Tirzepatide vs Albiglutide	0.87	0.51	1.48	0.87	0.000
**PIONEER 6**	Tirzepatide vs Efpeglenatide	1.12	0.62	2.04	1.12	0.000
**PIONEER 6**	Tirzepatide vs Exenatide	0.92	0.56	1.51	0.92	0.000
**PIONEER 6**	Tirzepatide vs Liraglutide	1.04	0.63	1.71	1.04	0.000

**PIONEER 6**	Tirzepatide vs Lixisenatide	0.83	0.49	1.39	0.83	0.000
**PIONEER 6**	Tirzepatide vs Semaglutide	0.95	0.61	1.49	1.01	-0.057
**REWIND**	Tirzepatide vs Placebo	NA	NA	NA	0.81	NA
**REWIND**	Tirzepatide vs Dulaglutide	NA	NA	NA	0.89	NA
**REWIND**	Tirzepatide vs Albiglutide	NA	NA	NA	0.87	NA
**REWIND**	Tirzepatide vs Efpeglenatide	NA	NA	NA	1.12	NA
**REWIND**	Tirzepatide vs Exenatide	NA	NA	NA	0.92	NA
**REWIND**	Tirzepatide vs Liraglutide	NA	NA	NA	1.04	NA
**REWIND**	Tirzepatide vs Lixisenatide	NA	NA	NA	0.83	NA
**REWIND**	Tirzepatide vs Semaglutide	NA	NA	NA	1.01	NA
**SOUL**	Tirzepatide vs Placebo	0.81	0.49	1.34	0.81	0.000
**SOUL**	Tirzepatide vs Dulaglutide	0.89	0.63	1.27	0.89	0.000
**SOUL**	Tirzepatide vs Albiglutide	0.87	0.46	1.66	0.87	0.000
**SOUL**	Tirzepatide vs Efpeglenatide	1.12	0.56	2.26	1.12	0.000
**SOUL**	Tirzepatide vs Exenatide	0.92	0.50	1.70	0.92	0.000
**SOUL**	Tirzepatide vs Liraglutide	1.04	0.56	1.93	1.04	0.000
**SOUL**	Tirzepatide vs Lixisenatide	0.83	0.44	1.57	0.83	0.000
**SOUL**	Tirzepatide vs Semaglutide	1.11	0.62	1.97	1.01	0.100
**SURPASS CVOT**	Tirzepatide vs Placebo	NA	NA	NA	0.81	NA
**SURPASS CVOT**	Tirzepatide vs Dulaglutide	NA	NA	NA	0.89	NA
**SURPASS CVOT**	Tirzepatide vs Albiglutide	NA	NA	NA	0.87	NA

**SURPASS CVOT**	Tirzepatide vs Efpeglenatide	NA	NA	NA	1.12	NA
**SURPASS CVOT**	Tirzepatide vs Exenatide	NA	NA	NA	0.92	NA
**SURPASS CVOT**	Tirzepatide vs Liraglutide	NA	NA	NA	1.04	NA
**SURPASS CVOT**	Tirzepatide vs Lixisenatide	NA	NA	NA	0.83	NA
**SURPASS CVOT**	Tirzepatide vs Semaglutide	NA	NA	NA	1.01	NA
**SUSTAIN 6**	Tirzepatide vs Placebo	0.81	0.43	1.52	0.81	0.000
**SUSTAIN 6**	Tirzepatide vs Dulaglutide	0.89	0.57	1.38	0.89	0.000
**SUSTAIN 6**	Tirzepatide vs Albiglutide	0.87	0.39	1.93	0.87	0.000
**SUSTAIN 6**	Tirzepatide vs Efpeglenatide	1.12	0.49	2.60	1.12	0.000
**SUSTAIN 6**	Tirzepatide vs Exenatide	0.92	0.43	1.99	0.92	0.000
**SUSTAIN 6**	Tirzepatide vs Liraglutide	1.04	0.48	2.25	1.04	0.000
**SUSTAIN 6**	Tirzepatide vs Lixisenatide	0.83	0.38	1.82	0.83	0.000
**SUSTAIN 6**	Tirzepatide vs Semaglutide	1.08	0.54	2.16	1.01	0.070

Supplemental Table S18. Leave-one-out sensitivity analysis at the agent-level for cardiovascular mortality comparing tirzepatide versus placebo, presenting hazard ratios with 95% confidence intervals after sequential removal of each individual trial.

<u>All-Cause Mortality</u>

Study Left Out	Tau-squared (τ^2)	I-squared (I^2)	Cochrane Q	Q p-value
**AMPLITUDE-O**	0.009	56.57	6.91	0.07
**ELIXA**	0.009	56.57	6.91	0.07
**EXSCEL**	0.009	56.57	6.91	0.07
**FLOW**	0.071	65.20	5.75	0.06
**Harmony Outcomes**	0.009	56.57	6.91	0.07
**LEADER**	0.009	56.57	6.91	0.07
**PIONEER 6**	0.001	16.19	2.39	0.30

**REWIND**	NA	NA	NA	NA
**SOUL**	0.062	62.96	5.40	0.07
**SURPASS CVOT**	0.009	56.57	6.91	0.07
**SUSTAIN 6**	0.025	64.90	5.70	0.06

Supplemental Table S19. Leave-one-out sensitivity analysis at the agent-level for all-cause mortality showing changes in between-study heterogeneity (τ^2 and I^2) following sequential exclusion of individual trials from the tirzepatide versus placebo comparison.

Study Left Out	Comparison	HR	Lower 95% CI	Upper 95% CI	Base HR	Delta HR
**AMPLITUDE-O**	Tirzepatide vs Placebo	0.76	0.56	1.03	0.76	0.000
**AMPLITUDE-O**	Tirzepatide vs Dulaglutide	0.84	0.68	1.04	0.84	0.000
**AMPLITUDE-O**	Tirzepatide vs Albiglutide	0.80	0.53	1.20	0.80	0.000
**AMPLITUDE-O**	Tirzepatide vs Efpeglenatide	NA	NA	NA	0.97	NA
**AMPLITUDE-O**	Tirzepatide vs Exenatide	0.88	0.60	1.28	0.88	0.000
**AMPLITUDE-O**	Tirzepatide vs Liraglutide	0.89	0.61	1.31	0.89	0.000
**AMPLITUDE-O**	Tirzepatide vs Lixisenatide	0.80	0.54	1.20	0.80	0.000
**AMPLITUDE-O**	Tirzepatide vs Semaglutide	0.89	0.63	1.25	0.89	0.000
**ELIXA**	Tirzepatide vs Placebo	0.76	0.56	1.03	0.76	0.000
**ELIXA**	Tirzepatide vs Dulaglutide	0.84	0.68	1.04	0.84	0.000
**ELIXA**	Tirzepatide vs Albiglutide	0.80	0.53	1.20	0.80	0.000
**ELIXA**	Tirzepatide vs Efpeglenatide	0.969	0.61	1.54	0.97	0.000
**ELIXA**	Tirzepatide vs Exenatide	0.879	0.60	1.28	0.88	0.000
**ELIXA**	Tirzepatide vs Liraglutide	0.889	0.61	1.31	0.89	0.000
**ELIXA**	Tirzepatide vs Lixisenatide	NA	NA	NA	0.80	NA

**ELIXA**	Tirzepatide vs Semaglutide	0.890	0.63	1.25	0.89	0.000
**EXSCEL**	Tirzepatide vs Placebo	0.756	0.56	1.03	0.76	0.000
**EXSCEL**	Tirzepatide vs Dulaglutide	0.840	0.68	1.04	0.84	0.000
**EXSCEL**	Tirzepatide vs Albiglutide	0.796	0.53	1.20	0.80	0.000
**EXSCEL**	Tirzepatide vs Efpeglenatide	0.969	0.61	1.54	0.97	0.000
**EXSCEL**	Tirzepatide vs Exenatide	NA	NA	NA	0.88	NA
**EXSCEL**	Tirzepatide vs Liraglutide	0.889	0.61	1.31	0.89	0.000
**EXSCEL**	Tirzepatide vs Lixisenatide	0.804	0.54	1.20	0.80	0.000
**EXSCEL**	Tirzepatide vs Semaglutide	0.890	0.63	1.25	0.89	0.000
**FLOW**	Tirzepatide vs Placebo	0.756	0.36	1.61	0.76	0.000
**FLOW**	Tirzepatide vs Dulaglutide	0.840	0.49	1.43	0.84	0.000
**FLOW**	Tirzepatide vs Albiglutide	0.796	0.31	2.03	0.80	0.000
**FLOW**	Tirzepatide vs Efpeglenatide	0.969	0.37	2.54	0.97	0.000
**FLOW**	Tirzepatide vs Exenatide	0.879	0.35	2.22	0.88	0.000
**FLOW**	Tirzepatide vs Liraglutide	0.889	0.35	2.25	0.89	0.000
**FLOW**	Tirzepatide vs Lixisenatide	0.804	0.32	2.05	0.80	0.000
**FLOW**	Tirzepatide vs Semaglutide	0.913	0.40	2.10	0.89	0.024
**Harmony Outcomes**	Tirzepatide vs Placebo	0.756	0.56	1.03	0.76	0.000
**Harmony Outcomes**	Tirzepatide vs Dulaglutide	0.840	0.68	1.04	0.84	0.000
**Harmony Outcomes**	Tirzepatide vs Albiglutide	NA	NA	NA	0.80	NA
**Harmony Outcomes**	Tirzepatide vs Efpeglenatide	0.969	0.61	1.54	0.97	0.000

**Harmony Outcomes**	Tirzepatide vs Exenatide	0.879	0.60	1.28	0.88	0.000
**Harmony Outcomes**	Tirzepatide vs Liraglutide	0.889	0.61	1.31	0.89	0.000
**Harmony Outcomes**	Tirzepatide vs Lixisenatide	0.804	0.54	1.20	0.80	0.000
**Harmony Outcomes**	Tirzepatide vs Semaglutide	0.890	0.63	1.25	0.89	0.000
**LEADER**	Tirzepatide vs Placebo	0.756	0.56	1.03	0.76	0.000
**LEADER**	Tirzepatide vs Dulaglutide	0.840	0.68	1.04	0.84	0.000
**LEADER**	Tirzepatide vs Albiglutide	0.796	0.53	1.20	0.80	0.000
**LEADER**	Tirzepatide vs Efpeglenatide	0.969	0.61	1.54	0.97	0.000
**LEADER**	Tirzepatide vs Exenatide	0.879	0.60	1.28	0.88	0.000
**LEADER**	Tirzepatide vs Liraglutide	NA	NA	NA	0.89	NA
**LEADER**	Tirzepatide vs Lixisenatide	0.804	0.54	1.20	0.80	0.000
**LEADER**	Tirzepatide vs Semaglutide	0.890	0.63	1.25	0.89	0.000
**PIONEER 6**	Tirzepatide vs Placebo	0.756	0.63	0.91	0.76	0.000
**PIONEER 6**	Tirzepatide vs Dulaglutide	0.840	0.74	0.96	0.84	0.000
**PIONEER 6**	Tirzepatide vs Albiglutide	0.796	0.60	1.05	0.80	0.000
**PIONEER 6**	Tirzepatide vs Efpeglenatide	0.969	0.68	1.38	0.97	0.000
**PIONEER 6**	Tirzepatide vs Exenatide	0.879	0.70	1.10	0.88	0.000
**PIONEER 6**	Tirzepatide vs Liraglutide	0.889	0.70	1.13	0.89	0.000
**PIONEER 6**	Tirzepatide vs Lixisenatide	0.804	0.61	1.05	0.80	0.000
**PIONEER 6**	Tirzepatide vs Semaglutide	0.855	0.69	1.06	0.89	-0.035
**REWIND**	Tirzepatide vs Placebo	NA	NA	NA	0.76	NA

**REWIND**	Tirzepatide vs Dulaglutide	NA	NA	NA	0.84	NA
**REWIND**	Tirzepatide vs Albiglutide	NA	NA	NA	0.80	NA
**REWIND**	Tirzepatide vs Efpeglenatide	NA	NA	NA	0.97	NA
**REWIND**	Tirzepatide vs Exenatide	NA	NA	NA	0.88	NA
**REWIND**	Tirzepatide vs Liraglutide	NA	NA	NA	0.89	NA
**REWIND**	Tirzepatide vs Lixisenatide	NA	NA	NA	0.80	NA
**REWIND**	Tirzepatide vs Semaglutide	NA	NA	NA	0.89	NA
**SOUL**	Tirzepatide vs Placebo	0.756	0.37	1.53	0.76	0.000
**SOUL**	Tirzepatide vs Dulaglutide	0.840	0.51	1.38	0.84	0.000
**SOUL**	Tirzepatide vs Albiglutide	0.796	0.33	1.92	0.80	0.000
**SOUL**	Tirzepatide vs Efpeglenatide	0.969	0.39	2.40	0.97	0.000
**SOUL**	Tirzepatide vs Exenatide	0.879	0.37	2.09	0.88	0.000
**SOUL**	Tirzepatide vs Liraglutide	0.889	0.37	2.12	0.89	0.000
**SOUL**	Tirzepatide vs Lixisenatide	0.804	0.33	1.94	0.80	0.000
**SOUL**	Tirzepatide vs Semaglutide	0.965	0.44	2.12	0.89	0.075
**SURPASS CVOT**	Tirzepatide vs Placebo	NA	NA	NA	0.76	NA
**SURPASS CVOT**	Tirzepatide vs Dulaglutide	NA	NA	NA	0.84	NA
**SURPASS CVOT**	Tirzepatide vs Albiglutide	NA	NA	NA	0.80	NA
**SURPASS CVOT**	Tirzepatide vs Efpeglenatide	NA	NA	NA	0.97	NA
**SURPASS CVOT**	Tirzepatide vs Exenatide	NA	NA	NA	0.88	NA
**SURPASS CVOT**	Tirzepatide vs Liraglutide	NA	NA	NA	0.89	NA

**SURPASS CVOT**	Tirzepatide vs Lixisenatide	NA	NA	NA	0.80	NA
**SURPASS CVOT**	Tirzepatide vs Semaglutide	NA	NA	NA	0.89	NA
**SUSTAIN 6**	Tirzepatide vs Placebo	0.756	0.47	1.21	0.76	0.000
**SUSTAIN 6**	Tirzepatide vs Dulaglutide	0.840	0.60	1.17	0.84	0.000
**SUSTAIN 6**	Tirzepatide vs Albiglutide	0.796	0.44	1.44	0.80	0.000
**SUSTAIN 6**	Tirzepatide vs Efpeglenatide	0.969	0.51	1.83	0.97	0.000
**SUSTAIN 6**	Tirzepatide vs Exenatide	0.879	0.50	1.56	0.88	0.000
**SUSTAIN 6**	Tirzepatide vs Liraglutide	0.889	0.50	1.58	0.89	0.000
**SUSTAIN 6**	Tirzepatide vs Lixisenatide	0.804	0.45	1.45	0.80	0.000
**SUSTAIN 6**	Tirzepatide vs Semaglutide	0.952	0.57	1.60	0.89	0.062

Supplemental Table S20. Leave-one-out sensitivity analysis at the agent-level for all-cause mortality comparing tirzepatide versus placebo, presenting hazard ratios with 95% confidence intervals after sequential removal of each individual trial.

Non-Fatal Myocardial Infarction

Study Left Out	Tau-squared (τ^2)	I-squared (I^2)	Cochrane Q	Q p-value
**AMPLITUDE-O**	0.000	8.21	3.27	0.35
**ELIXA**	0.000	8.21	3.27	0.35
**EXSCEL**	0.000	8.21	3.27	0.35
**FLOW**	0.011	38.50	3.25	0.20
**LEADER**	0.000	8.21	3.27	0.35
**PIONEER 6**	0.000	0.00	0.14	0.93
**REWIND**	NA	NA	NA	NA
**SOUL**	0.001	17.87	2.44	0.30
**SURPASS CVOT**	0.000	8.21	3.27	0.35
**SUSTAIN 6**	0.013	36.88	3.17	0.21

Supplemental Table S21. Leave-one-out sensitivity analysis at the agent-level for non-fatal myocardial infarction showing changes in between-study heterogeneity (τ^2 and I^2) following sequential exclusion of individual trials from the tirzepatide versus placebo comparison.

Study Left Out	Comparison	HR	Lower 95% CI	Upper 95% CI	Base HR	Delta HR
**AMPLITUDE-O**	Tirzepatide vs Placebo	0.83	0.65	1.05	0.83	0.000
**AMPLITUDE-O**	Tirzepatide vs Dulaglutide	0.86	0.74	1.00	0.86	0.000
**AMPLITUDE-O**	Tirzepatide vs Efpeglenatide	NA	NA	NA	1.06	NA
**AMPLITUDE-O**	Tirzepatide vs Exenatide	0.85	0.65	1.12	0.85	0.000
**AMPLITUDE-O**	Tirzepatide vs Liraglutide	0.94	0.70	1.26	0.94	0.000
**AMPLITUDE-O**	Tirzepatide vs Lixisenatide	0.80	0.60	1.08	0.80	0.000
**AMPLITUDE-O**	Tirzepatide vs Semaglutide	1.06	0.79	1.40	1.06	0.000
**ELIXA**	Tirzepatide vs Placebo	0.83	0.65	1.05	0.83	0.000
**ELIXA**	Tirzepatide vs Dulaglutide	0.86	0.74	1.00	0.86	0.000
**ELIXA**	Tirzepatide vs Efpeglenatide	1.06	0.69	1.62	1.06	0.000
**ELIXA**	Tirzepatide vs Exenatide	0.85	0.65	1.12	0.85	0.000
**ELIXA**	Tirzepatide vs Liraglutide	0.94	0.70	1.26	0.94	0.000
**ELIXA**	Tirzepatide vs Lixisenatide	NA	NA	NA	0.80	NA
**ELIXA**	Tirzepatide vs Semaglutide	1.06	0.79	1.40	1.06	0.000
**EXSCEL**	Tirzepatide vs Placebo	0.83	0.65	1.05	0.83	0.000
**EXSCEL**	Tirzepatide vs Dulaglutide	0.86	0.74	1.00	0.86	0.000
**EXSCEL**	Tirzepatide vs Efpeglenatide	1.06	0.69	1.62	1.06	0.000
**EXSCEL**	Tirzepatide vs Exenatide	NA	NA	NA	0.85	NA
**EXSCEL**	Tirzepatide vs Liraglutide	0.94	0.70	1.26	0.94	0.000
**EXSCEL**	Tirzepatide vs Lixisenatide	0.80	0.60	1.08	0.80	0.000
**EXSCEL**	Tirzepatide vs Semaglutide	1.06	0.79	1.40	1.06	0.000
**FLOW**	Tirzepatide vs Placebo	0.83	0.57	1.20	0.83	0.000
**FLOW**	Tirzepatide vs Dulaglutide	0.86	0.67	1.11	0.86	0.000
**FLOW**	Tirzepatide vs Efpeglenatide	1.06	0.61	1.84	1.06	0.000
**FLOW**	Tirzepatide vs Exenatide	0.85	0.54	1.33	0.85	0.000
**FLOW**	Tirzepatide vs Liraglutide	0.94	0.59	1.48	0.94	0.000

**FLOW**	Tirzepatide vs Lixisenatide	0.80	0.51	1.27	0.80	0.000
**FLOW**	Tirzepatide vs Semaglutide	1.03	0.67	1.59	1.06	-0.023
**LEADER**	Tirzepatide vs Placebo	0.83	0.65	1.05	0.83	0.000
**LEADER**	Tirzepatide vs Dulaglutide	0.86	0.74	1.00	0.86	0.000
**LEADER**	Tirzepatide vs Efpeglenatide	1.06	0.69	1.62	1.06	0.000
**LEADER**	Tirzepatide vs Exenatide	0.85	0.65	1.12	0.85	0.000
**LEADER**	Tirzepatide vs Liraglutide	NA	NA	NA	0.94	NA
**LEADER**	Tirzepatide vs Lixisenatide	0.80	0.60	1.08	0.80	0.000
**LEADER**	Tirzepatide vs Semaglutide	1.06	0.79	1.40	1.06	0.000
**PIONEER 6**	Tirzepatide vs Placebo	0.83	0.65	1.05	0.83	0.000
**PIONEER 6**	Tirzepatide vs Dulaglutide	0.86	0.74	1.00	0.86	0.000
**PIONEER 6**	Tirzepatide vs Efpeglenatide	1.06	0.69	1.62	1.06	0.000
**PIONEER 6**	Tirzepatide vs Exenatide	0.85	0.65	1.12	0.85	0.000
**PIONEER 6**	Tirzepatide vs Liraglutide	0.94	0.70	1.26	0.94	0.000
**PIONEER 6**	Tirzepatide vs Lixisenatide	0.80	0.60	1.08	0.80	0.000
**PIONEER 6**	Tirzepatide vs Semaglutide	1.10	0.83	1.47	1.06	0.046
**REWIND**	Tirzepatide vs Placebo	NA	NA	NA	0.83	NA
**REWIND**	Tirzepatide vs Dulaglutide	NA	NA	NA	0.86	NA
**REWIND**	Tirzepatide vs Efpeglenatide	NA	NA	NA	1.06	NA
**REWIND**	Tirzepatide vs Exenatide	NA	NA	NA	0.85	NA
**REWIND**	Tirzepatide vs Liraglutide	NA	NA	NA	0.94	NA
**REWIND**	Tirzepatide vs Lixisenatide	NA	NA	NA	0.80	NA
**REWIND**	Tirzepatide vs Semaglutide	NA	NA	NA	1.06	NA
**SOUL**	Tirzepatide vs Placebo	0.83	0.64	1.07	0.83	0.000
**SOUL**	Tirzepatide vs Dulaglutide	0.86	0.73	1.01	0.86	0.000
**SOUL**	Tirzepatide vs Efpeglenatide	1.06	0.69	1.63	1.06	0.000
**SOUL**	Tirzepatide vs Exenatide	0.85	0.64	1.14	0.85	0.000

**SOUL**	Tirzepatide vs Liraglutide	0.94	0.69	1.27	0.94	0.000
**SOUL**	Tirzepatide vs Lixisenatide	0.80	0.59	1.09	0.80	0.000
**SOUL**	Tirzepatide vs Semaglutide	0.97	0.69	1.37	1.06	-0.085
**SURPASS CVOT**	Tirzepatide vs Placebo	NA	NA	NA	0.83	NA
**SURPASS CVOT**	Tirzepatide vs Dulaglutide	NA	NA	NA	0.86	NA
**SURPASS CVOT**	Tirzepatide vs Efpeglenatide	NA	NA	NA	1.06	NA
**SURPASS CVOT**	Tirzepatide vs Exenatide	NA	NA	NA	0.85	NA
**SURPASS CVOT**	Tirzepatide vs Liraglutide	NA	NA	NA	0.94	NA
**SURPASS CVOT**	Tirzepatide vs Lixisenatide	NA	NA	NA	0.80	NA
**SURPASS CVOT**	Tirzepatide vs Semaglutide	NA	NA	NA	1.06	NA
**SUSTAIN 6**	Tirzepatide vs Placebo	0.83	0.55	1.23	0.83	0.000
**SUSTAIN 6**	Tirzepatide vs Dulaglutide	0.86	0.66	1.13	0.86	0.000
**SUSTAIN 6**	Tirzepatide vs Efpeglenatide	1.06	0.60	1.88	1.06	0.000
**SUSTAIN 6**	Tirzepatide vs Exenatide	0.85	0.53	1.37	0.85	0.000
**SUSTAIN 6**	Tirzepatide vs Liraglutide	0.94	0.58	1.53	0.94	0.000
**SUSTAIN 6**	Tirzepatide vs Lixisenatide	0.80	0.49	1.31	0.80	0.000
**SUSTAIN 6**	Tirzepatide vs Semaglutide	1.01	0.64	1.59	1.06	-0.047

Supplemental Table S22. Leave-one-out sensitivity analysis at the agent-level for non-fatal myocardial infarction comparing tirzepatide versus placebo, presenting hazard ratios with 95% confidence intervals after sequential removal of each individual trial.

Non-Fatal Stroke

**Study Left Out**	**Tau-squared (τ²)**	**I-squared (I²)**	**Cochrane Q**	**Q p-value**
**AMPLITUDE-O**	0.040	44.81	5.44	0.14
**ELIXA**	0.040	44.81	5.44	0.14
**EXSCEL**	0.040	44.81	5.44	0.14
**FLOW**	0.013	0.00	1.93	0.38
**LEADER**	0.040	44.81	5.44	0.14
**PIONEER 6**	0.059	61.44	5.19	0.07

**REWIND**	NA	NA	NA	NA
**SOUL**	0.103	63.02	5.41	0.07
**SURPASS CVOT**	0.040	44.81	5.44	0.14
**SUSTAIN 6**	0.015	22.45	2.58	0.28

Supplemental Table S23. Leave-one-out sensitivity analysis at the agent-level for non-fatal stroke showing changes in between-study heterogeneity (τ^2 and I^2) following sequential exclusion of individual trials from the tirzepatide versus placebo comparison.

Study Left Out	Comparison	HR	Lower 95% CI	Upper 95% CI	Base HR	Delta HR
**AMPLITUDE-O**	Tirzepatide vs Placebo	0.69	0.37	1.29	0.69	0.000
**AMPLITUDE-O**	Tirzepatide vs Dulaglutide	0.91	0.59	1.40	0.91	0.000
**AMPLITUDE-O**	Tirzepatide vs Efpeglenatide	NA	NA	NA	0.86	NA
**AMPLITUDE-O**	Tirzepatide vs Exenatide	0.81	0.38	1.74	0.81	0.000
**AMPLITUDE-O**	Tirzepatide vs Liraglutide	0.78	0.36	1.67	0.78	0.000
**AMPLITUDE-O**	Tirzepatide vs Lixisenatide	0.62	0.27	1.39	0.62	0.000
**AMPLITUDE-O**	Tirzepatide vs Semaglutide	0.79	0.40	1.57	0.79	0.000
**ELIXA**	Tirzepatide vs Placebo	0.69	0.37	1.29	0.69	0.000
**ELIXA**	Tirzepatide vs Dulaglutide	0.91	0.59	1.40	0.91	0.000
**ELIXA**	Tirzepatide vs Efpeglenatide	0.86	0.35	2.11	0.86	0.000
**ELIXA**	Tirzepatide vs Exenatide	0.814	0.38	1.74	0.81	0.000
**ELIXA**	Tirzepatide vs Liraglutide	0.777	0.36	1.67	0.78	0.000
**ELIXA**	Tirzepatide vs Lixisenatide	NA	NA	NA	0.62	NA
**ELIXA**	Tirzepatide vs Semaglutide	0.792	0.40	1.57	0.79	0.000
**EXSCEL**	Tirzepatide vs Placebo	0.692	0.37	1.29	0.69	0.000
**EXSCEL**	Tirzepatide vs Dulaglutide	0.910	0.59	1.40	0.91	0.000
**EXSCEL**	Tirzepatide vs Efpeglenatide	0.865	0.35	2.11	0.86	0.000
**EXSCEL**	Tirzepatide vs Exenatide	NA	NA	NA	0.81	NA
**EXSCEL**	Tirzepatide vs Liraglutide	0.777	0.36	1.67	0.78	0.000
**EXSCEL**	Tirzepatide vs Lixisenatide	0.618	0.27	1.39	0.62	0.000

**EXSCEL**	Tirzepatide vs Semaglutide	0.792	0.40	1.57	0.79	0.000
**FLOW**	Tirzepatide vs Placebo	0.692	0.45	1.06	0.69	0.000
**FLOW**	Tirzepatide vs Dulaglutide	0.910	0.68	1.21	0.91	0.000
**FLOW**	Tirzepatide vs Efpeglenatide	0.865	0.43	1.74	0.86	0.000
**FLOW**	Tirzepatide vs Exenatide	0.814	0.48	1.37	0.81	0.000
**FLOW**	Tirzepatide vs Liraglutide	0.777	0.46	1.32	0.78	0.000
**FLOW**	Tirzepatide vs Lixisenatide	0.618	0.34	1.12	0.62	0.000
**FLOW**	Tirzepatide vs Semaglutide	0.876	0.53	1.44	0.79	0.084
**LEADER**	Tirzepatide vs Placebo	0.692	0.37	1.29	0.69	0.000
**LEADER**	Tirzepatide vs Dulaglutide	0.910	0.59	1.40	0.91	0.000
**LEADER**	Tirzepatide vs Efpeglenatide	0.865	0.35	2.11	0.86	0.000
**LEADER**	Tirzepatide vs Exenatide	0.814	0.38	1.74	0.81	0.000
**LEADER**	Tirzepatide vs Liraglutide	NA	NA	NA	0.78	NA
**LEADER**	Tirzepatide vs Lixisenatide	0.618	0.27	1.39	0.62	0.000
**LEADER**	Tirzepatide vs Semaglutide	0.792	0.40	1.57	0.79	0.000
**PIONEER 6**	Tirzepatide vs Placebo	0.692	0.33	1.44	0.69	0.000
**PIONEER 6**	Tirzepatide vs Dulaglutide	0.910	0.55	1.51	0.91	0.000
**PIONEER 6**	Tirzepatide vs Efpeglenatide	0.865	0.32	2.37	0.86	0.000
**PIONEER 6**	Tirzepatide vs Exenatide	0.814	0.33	1.99	0.81	0.000
**PIONEER 6**	Tirzepatide vs Liraglutide	0.777	0.32	1.91	0.78	0.000
**PIONEER 6**	Tirzepatide vs Lixisenatide	0.618	0.24	1.58	0.62	0.000
**PIONEER 6**	Tirzepatide vs Semaglutide	0.778	0.35	1.74	0.79	-0.013
**REWIND**	Tirzepatide vs Placebo	NA	NA	NA	0.69	NA
**REWIND**	Tirzepatide vs Dulaglutide	NA	NA	NA	0.91	NA
**REWIND**	Tirzepatide vs Efpeglenatide	NA	NA	NA	0.86	NA
**REWIND**	Tirzepatide vs Exenatide	NA	NA	NA	0.81	NA
**REWIND**	Tirzepatide vs Liraglutide	NA	NA	NA	0.78	NA

**REWIND**	Tirzepatide vs Lixisenatide	NA	NA	NA	0.62	NA
**REWIND**	Tirzepatide vs Semaglutide	NA	NA	NA	0.79	NA
**SOUL**	Tirzepatide vs Placebo	0.692	0.27	1.76	0.69	0.000
**SOUL**	Tirzepatide vs Dulaglutide	0.910	0.47	1.75	0.91	0.000
**SOUL**	Tirzepatide vs Efpeglenatide	0.865	0.25	2.97	0.86	0.000
**SOUL**	Tirzepatide vs Exenatide	0.814	0.26	2.55	0.81	0.000
**SOUL**	Tirzepatide vs Liraglutide	0.777	0.25	2.45	0.78	0.000
**SOUL**	Tirzepatide vs Lixisenatide	0.618	0.19	2.01	0.62	0.000
**SOUL**	Tirzepatide vs Semaglutide	0.814	0.29	2.32	0.79	0.022
**SURPASS CVOT**	Tirzepatide vs Placebo	NA	NA	NA	0.69	NA
**SURPASS CVOT**	Tirzepatide vs Dulaglutide	NA	NA	NA	0.91	NA
**SURPASS CVOT**	Tirzepatide vs Efpeglenatide	NA	NA	NA	0.86	NA
**SURPASS CVOT**	Tirzepatide vs Exenatide	NA	NA	NA	0.81	NA
**SURPASS CVOT**	Tirzepatide vs Liraglutide	NA	NA	NA	0.78	NA
**SURPASS CVOT**	Tirzepatide vs Lixisenatide	NA	NA	NA	0.62	NA
**SURPASS CVOT**	Tirzepatide vs Semaglutide	NA	NA	NA	0.79	NA
**SUSTAIN 6**	Tirzepatide vs Placebo	0.692	0.44	1.08	0.69	0.000
**SUSTAIN 6**	Tirzepatide vs Dulaglutide	0.910	0.67	1.23	0.91	0.000
**SUSTAIN 6**	Tirzepatide vs Efpeglenatide	0.865	0.42	1.77	0.86	0.000
**SUSTAIN 6**	Tirzepatide vs Exenatide	0.814	0.47	1.40	0.81	0.000
**SUSTAIN 6**	Tirzepatide vs Liraglutide	0.777	0.45	1.35	0.78	0.000
**SUSTAIN 6**	Tirzepatide vs Lixisenatide	0.618	0.33	1.14	0.62	0.000
**SUSTAIN 6**	Tirzepatide vs Semaglutide	0.720	0.43	1.20	0.79	-0.072

Supplemental Table S24. Leave-one-out sensitivity analysis at the agent-level for non-fatal stroke comparing tirzepatide versus placebo, presenting hazard ratios with 95% confidence intervals after sequential removal of each individual trial.

Certainty of evidence (GRADE) using Confidence in Network Meta-Analysis (CINeMA) for the class-level network meta-analysis

Outcome	Comparison (HR, 95% CI)	Direct comps (n)	Within-study bias	Reporting bias	Indirectness	Imprecision	Heterogeneity	Incoherence	Confidence rating
MACE	Tirzepatide vs placebo (HR 0.79, 0.69-0.91)	0	No concerns	No concerns	Some concerns (indirect-only; potential intransitivity)	No concerns	Some concerns (I^2 = 31.38%)	Not assessable*	Low
MACE	GLP-1RA vs placebo (HR 0.86, 0.82-0.91)	10	No concerns	No concerns	No concerns	No concerns	Some concerns (I^2 = 31.38%)	Not assessable*	Moderate
CV mortality	Tirzepatide vs placebo (HR 0.77, 0.66-0.90)	0	No concerns	No concerns	Some concerns (indirect-only; potential intransitivity)	No concerns	No concerns (I^2 = 23.5%)	Not assessable*	Low
CV mortality	GLP-1RA vs placebo (HR 0.87, 0.81-0.92)	10	No concerns	No concerns	No concerns	No concerns	No concerns (I^2 = 23.5%)	Not assessable*	High
All-cause mortality	Tirzepatide vs placebo (HR 0.74, 0.65-0.83)	0	No concerns	No concerns	Some concerns (indirect-only; potential intransitivity)	No concerns	No concerns (I^2 = 2.76%)	Not assessable*	Low
All-cause mortality	GLP-1RA vs placebo (HR 0.88, 0.83-0.92)	10	No concerns	No concerns	No concerns	No concerns	No concerns (I^2 = 2.76%)	Not assessable*	High
Non-fatal MI	Tirzepatide vs placebo (HR 0.77, 0.61-0.97)	0	No concerns	No concerns	Some concerns (indirect-only; potential intransitivity)	Some concerns (CI close to 1; relatively wide)	Some concerns (I^2 = 31.09%)	Not assessable*	Low
Non-fatal MI	GLP-1RA vs placebo (HR 0.90, 0.82-0.98)	9	No concerns	No concerns	No concerns	Some concerns (CI close to 1)	Some concerns (I^2 = 31.09%)	Not assessable*	Moderate
Non-fatal stroke	Tirzepatide vs placebo (HR 0.79, 0.64-0.97)	0	No concerns	No concerns	Some concerns (indirect-only; potential intransitivity)	Some concerns (CI close to 1)	No concerns (I^2 = 12.85%)	Not assessable*	Low
Non-fatal stroke	GLP-1RA vs placebo (HR 0.87, 0.79-0.95)	9	No concerns	No concerns	No concerns	No concerns	No concerns (I^2 = 12.85%)	Not assessable*	High

Supplemental Table S25: Certainty of evidence for class-level network estimates was assessed using the CINeMA framework across key outcomes, with domain judgments and overall confidence ratings reported for each comparison. * Incoherence was not assessable because there were insufficient data to contrast direct and indirect evidence for the same comparison.

Certainty of evidence (GRADE) using Confidence in Network Meta-Analysis (CINeMA) for the agent-level network meta-analysis

Outcome	Comparison (HR, 95% CI)	Direct comps (n)	Within-study bias	Reporting bias	Indirectness	Imprecision	Heterogeneity	Incoherence	Confidence rating
MACE	Tirzepatide vs placebo (0.81, 0.70–0.94)	0	No concerns	No concerns	Major concerns (indirect-only)	No concerns	No concerns (I^2 = 0%)	Not assessable*	Low
MACE	Albiglutide vs placebo (0.78, 0.68–0.90)	1	No concerns	No concerns	No concerns	No concerns	No concerns (I^2 = 0%)	Not assessable*	High
MACE	Dulaglutide vs placebo (0.88, 0.79–0.98)	1	No concerns	No concerns	No concerns	No concerns	No concerns (I^2 = 0%)	Not assessable*	High
MACE	Efpeglenatide vs placebo (0.73, 0.58–0.92)	1	No concerns	No concerns	No concerns	Some concerns (wide CI)	No concerns (I^2 = 0%)	Not assessable*	Moderate
MACE	Exenatide vs placebo (0.91, 0.83–1.00)	1	No concerns	No concerns	No concerns	Some concerns (borderline CI)	No concerns (I^2 = 0%)	Not assessable*	Moderate
MACE	Liraglutide vs placebo (0.87, 0.78–0.97)	1	No concerns	No concerns	No concerns	No concerns	No concerns (I^2 = 0%)	Not assessable*	High
MACE	Lixisenatide vs placebo (1.02, 0.89–1.17)	1	No concerns	No concerns	No concerns	Some concerns (CI crosses 1)	No concerns (I^2 = 0%)	Not assessable*	Moderate
MACE	Semaglutide vs placebo (0.83, 0.76–0.91)	4	No concerns	No concerns	No concerns	No concerns	No concerns (I^2 = 0%)	Not assessable*	High
MACE	Tirzepatide vs Albiglutide (1.04, 0.85–1.27)	0	No concerns	No concerns	Major concerns (indirect-only)	Some concerns (CI crosses 1)	No concerns (I^2 = 0%)	Not assessable*	Very low
MACE	Tirzepatide vs Dulaglutide (0.92, 0.83–1.07)	1	No concerns	No concerns	No concerns	Some concerns (CI crosses 1)	No concerns (I^2 = 0%)	Not assessable*	Low
MACE	Tirzepatide vs Efpeglenatide (1.11, 0.84–1.46)	0	No concerns	No concerns	Major concerns (indirect-only)	Some concerns (CI crosses 1)	No concerns (I^2 = 0%)	Not assessable*	Very low
MACE	Tirzepatide vs Exenatide (0.89, 0.75–1.06)	0	No concerns	No concerns	Major concerns (indirect-only)	Some concerns (CI crosses 1)	No concerns (I^2 = 0%)	Not assessable*	Very low
MACE	Tirzepatide vs Liraglutide (0.93, 0.78–1.12)	0	No concerns	No concerns	Major concerns (indirect-only)	Some concerns (CI crosses 1)	No concerns (I^2 = 0%)	Not assessable*	Very low

MACE	Tirzepatide vs Lixisenatide (0.79, 0.65–0.97)	0	No concerns	No concerns	Major concerns (indirect-only)	No concerns	No concerns (I^2 = 0%)	Not assessable*	Low
MACE	Tirzepatide vs Semaglutide (0.97, 0.82–1.15)	0	No concerns	No concerns	Major concerns (indirect-only)	Some concerns (CI crosses 1)	No concerns (I^2 = 0%)	Not assessable*	Very low
CV mortality	Tirzepatide vs placebo (0.81, 0.50–1.32)	0	No concerns	No concerns	Major concerns (indirect-only)	Some concerns (wide CI)	Some concerns (I^2 = 58.85%)	Not assessable*	Very low
CV mortality	Albiglutide vs placebo (0.93, 0.62–1.39)	1	No concerns	No concerns	No concerns	Some concerns (wide CI)	Some concerns (I^2 = 58.85%)	Not assessable*	Low
CV mortality	Dulaglutide vs placebo (0.91, 0.64–1.29)	1	No concerns	No concerns	No concerns	Some concerns (wide CI)	Some concerns (I^2 = 58.85%)	Not assessable*	Low
CV mortality	Efpeglenatide vs placebo (0.72, 0.45–1.16)	1	No concerns	No concerns	No concerns	Some concerns (wide CI)	Some concerns (I^2 = 58.85%)	Not assessable*	Low
CV mortality	Exenatide vs placebo (0.88, 0.62–1.25)	1	No concerns	No concerns	No concerns	Some concerns (wide CI)	Some concerns (I^2 = 58.85%)	Not assessable*	Low
CV mortality	Liraglutide vs placebo (0.78, 0.54–1.12)	1	No concerns	No concerns	No concerns	Some concerns (CI crosses 1)	Some concerns (I^2 = 58.85%)	Not assessable*	Low
CV mortality	Lixisenatide vs placebo (0.98, 0.66–1.45)	1	No concerns	No concerns	No concerns	Some concerns (wide CI)	Some concerns (I^2 = 58.85%)	Not assessable*	Low
CV mortality	Semaglutide vs placebo (0.81, 0.65–1.00)	4	No concerns	No concerns	No concerns	Some concerns (borderline CI)	Some concerns (I^2 = 58.85%)	Not assessable*	Low
CV mortality	Tirzepatide vs Albiglutide (1.15, 0.61–2.16)	0	No concerns	No concerns	Major concerns (indirect-only)	Major concerns (very wide CI)	Some concerns (I^2 = 58.85%)	Not assessable*	Very low
CV mortality	Tirzepatide vs Dulaglutide (1.12, 0.80–1.59)	1	No concerns	No concerns	No concerns	Some concerns (CI crosses 1)	Some concerns (I^2 = 58.85%)	Not assessable*	Very low
CV mortality	Tirzepatide vs Efpeglenatide (0.89, 0.45–1.77)	0	No concerns	No concerns	Major concerns (indirect-only)	Major concerns (very wide CI)	Some concerns (I^2 = 58.85%)	Not assessable*	Very low
CV mortality	Tirzepatide vs Exenatide (0.92, 0.50–1.68)	0	No concerns	No concerns	Major concerns (indirect-only)	Major concerns (very wide CI)	Some concerns (I^2 = 58.85%)	Not assessable*	Very low
CV mortality	Tirzepatide vs Liraglutide (1.04, 0.56–1.91)	0	No concerns	No concerns	Major concerns (indirect-only)	Major concerns (very wide CI)	Some concerns (I^2 = 58.85%)	Not assessable*	Very low

CV mortality	Tirzepatide vs Lixisenatide (0.83, 0.44–1.55)	0	No concerns	No concerns	Major concerns (indirect-only)	Major concerns (very wide CI)	Some concerns (I² = 58.85%)	Not assessable*	Very low
CV mortality	Tirzepatide vs Semaglutide (1.01, 0.59–1.72)	0	No concerns	No concerns	Major concerns (indirect-only)	Major concerns (very wide CI)	Some concerns (I² = 58.85%)	Not assessable*	Very low
All-cause mortality	Tirzepatide vs placebo (0.76, 0.56–1.03)	0	No concerns	No concerns	Major concerns (indirect-only)	Some concerns (CI crosses 1)	Some concerns (I² = 56.57%)	Not assessable*	Very low
All-cause mortality	Albiglutide vs placebo (0.95, 0.73–1.24)	1	No concerns	No concerns	No concerns	Some concerns (CI crosses 1)	Some concerns (I² = 56.57%)	Not assessable*	Low
All-cause mortality	Dulaglutide vs placebo (0.90, 0.72–1.12)	1	No concerns	No concerns	No concerns	Some concerns (CI crosses 1)	Some concerns (I² = 56.57%)	Not assessable*	Low
All-cause mortality	Efpeglenatide vs placebo (0.78, 0.55–1.11)	1	No concerns	No concerns	No concerns	Some concerns (CI crosses 1)	Some concerns (I² = 56.57%)	Not assessable*	Low
All-cause mortality	Exenatide vs placebo (0.86, 0.69–1.07)	1	No concerns	No concerns	No concerns	Some concerns (CI crosses 1)	Some concerns (I² = 56.57%)	Not assessable*	Low
All-cause mortality	Liraglutide vs placebo (0.85, 0.68–1.07)	1	No concerns	No concerns	No concerns	Some concerns (CI crosses 1)	Some concerns (I² = 56.57%)	Not assessable*	Low
All-cause mortality	Lixisenatide vs placebo (0.94, 0.72–1.22)	1	No concerns	No concerns	No concerns	Some concerns (CI crosses 1)	Some concerns (I² = 56.57%)	Not assessable*	Low
All-cause mortality	Semaglutide vs placebo (0.85, 0.73–0.99)	4	No concerns	No concerns	No concerns	Some concerns (borderline CI)	Some concerns (I² = 56.57%)	Not assessable*	Low
All-cause mortality	Tirzepatide vs Albiglutide (0.80, 0.53–1.20)	0	No concerns	No concerns	Major concerns (indirect-only)	Major concerns (wide CI)	Some concerns (I² = 56.57%)	Not assessable*	Very low
All-cause mortality	Tirzepatide vs Dulaglutide (0.84, 0.68–1.04)	1	No concerns	No concerns	No concerns	Some concerns (CI crosses 1)	Some concerns (I² = 56.57%)	Not assessable*	Very low
All-cause mortality	Tirzepatide vs Efpeglenatide (0.97, 0.61–1.54)	0	No concerns	No concerns	Major concerns (indirect-only)	Major concerns (wide CI)	Some concerns (I² = 56.57%)	Not assessable*	Very low
All-cause mortality	Tirzepatide vs Exenatide (0.88, 0.60–1.28)	0	No concerns	No concerns	Major concerns (indirect-only)	Major concerns (wide CI)	Some concerns (I² = 56.57%)	Not assessable*	Very low
All-cause mortality	Tirzepatide vs Liraglutide (0.89, 0.61–1.31)	0	No concerns	No concerns	Major concerns (indirect-only)	Major concerns (wide CI)	Some concerns (I² = 56.57%)	Not assessable*	Very low

All-cause mortality	Tirzepatide vs Lixisenatide (0.80, 0.54–1.20)	0	No concerns	No concerns	Major concerns (indirect-only)	Major concerns (wide CI)	Some concerns (I^2 = 56.57%)	Not assessable*	Very low
All-cause mortality	Tirzepatide vs Semaglutide (0.89, 0.63–1.25)	0	No concerns	No concerns	Major concerns (indirect-only)	Some concerns (CI crosses 1)	Some concerns (I^2 = 56.57%)	Not assessable*	Very low
Non-fatal MI	Tirzepatide vs placebo (0.83, 0.65–1.05)	0	No concerns	No concerns	Major concerns (indirect-only)	Some concerns (CI crosses 1)	No concerns (I^2 = 8.21%)	Not assessable*	Very low
Non-fatal MI	Dulaglutide vs placebo (0.96, 0.79–1.16)	1	No concerns	No concerns	No concerns	Some concerns (CI crosses 1)	No concerns (I^2 = 8.21%)	Not assessable*	Moderate
Non-fatal MI	Efpeglenatide vs placebo (0.78, 0.55–1.10)	1	No concerns	No concerns	No concerns	Some concerns (CI crosses 1)	No concerns (I^2 = 8.21%)	Not assessable*	Moderate
Non-fatal MI	Exenatide vs placebo (0.97, 0.85–1.10)	1	No concerns	No concerns	No concerns	Some concerns (CI crosses 1)	No concerns (I^2 = 8.21%)	Not assessable*	Moderate
Non-fatal MI	Liraglutide vs placebo (0.88, 0.75–1.03)	1	No concerns	No concerns	No concerns	Some concerns (CI crosses 1)	No concerns (I^2 = 8.21%)	Not assessable*	Moderate
Non-fatal MI	Lixisenatide vs placebo (1.03, 0.87–1.22)	1	No concerns	No concerns	No concerns	Some concerns (CI crosses 1)	No concerns (I^2 = 8.21%)	Not assessable*	Moderate
Non-fatal MI	Semaglutide vs placebo (0.78, 0.68–0.91)	4	No concerns	No concerns	No concerns	No concerns	No concerns (I^2 = 8.21%)	Not assessable*	High
Non-fatal MI	Tirzepatide vs Dulaglutide (0.86, 0.74–1.00)	1	No concerns	No concerns	No concerns	Some concerns (borderline CI)	No concerns (I^2 = 8.21%)	Not assessable*	Low
Non-fatal MI	Tirzepatide vs Efpeglenatide (1.06, 0.69–1.62)	0	No concerns	No concerns	Major concerns (indirect-only)	Major concerns (wide CI)	No concerns (I^2 = 8.21%)	Not assessable*	Very low
Non-fatal MI	Tirzepatide vs Exenatide (0.85, 0.65–1.12)	0	No concerns	No concerns	Major concerns (indirect-only)	Some concerns (CI crosses 1)	No concerns (I^2 = 8.21%)	Not assessable*	Very low
Non-fatal MI	Tirzepatide vs Liraglutide (0.94, 0.70–1.26)	0	No concerns	No concerns	Major concerns (indirect-only)	Some concerns (CI crosses 1)	No concerns (I^2 = 8.21%)	Not assessable*	Very low
Non-fatal MI	Tirzepatide vs Lixisenatide (0.80, 0.60–1.08)	0	No concerns	No concerns	Major concerns (indirect-only)	Some concerns (CI crosses 1)	No concerns (I^2 = 8.21%)	Not assessable*	Very low
Non-fatal MI	Tirzepatide vs Semaglutide (1.06, 0.79–1.40)	0	No concerns	No concerns	Major concerns (indirect-only)	Some concerns (CI crosses 1)	No concerns (I^2 = 8.21%)	Not assessable*	Very low

Non-fatal stroke	Tirzepatide vs placebo (0.69, 0.37–1.29)	0	No concerns	No concerns	Major concerns (indirect-only)	Major concerns (very wide CI)	Some concerns ($I^2 = 44.81\%$)	Not assessable*	Very low
Non-fatal stroke	Dulaglutide vs placebo (0.76, 0.49–1.19)	1	No concerns	No concerns	No concerns	Some concerns (CI crosses 1)	Some concerns ($I^2 = 44.81\%$)	Not assessable*	Moderate
Non-fatal stroke	Efpeglenatide vs placebo (0.80, 0.42–1.52)	1	No concerns	No concerns	No concerns	Major concerns (very wide CI)	Some concerns ($I^2 = 44.81\%$)	Not assessable*	Low
Non-fatal stroke	Exenatide vs placebo (0.85, 0.55–1.31)	1	No concerns	No concerns	No concerns	Some concerns (CI crosses 1)	Some concerns ($I^2 = 44.81\%$)	Not assessable*	Moderate
Non-fatal stroke	Liraglutide vs placebo (0.89, 0.57–1.39)	1	No concerns	No concerns	No concerns	Some concerns (CI crosses 1)	Some concerns ($I^2 = 44.81\%$)	Not assessable*	Moderate
Non-fatal stroke	Lixisenatide vs placebo (1.12, 0.66–1.89)	1	No concerns	No concerns	No concerns	Major concerns (very wide CI)	Some concerns ($I^2 = 44.81\%$)	Not assessable*	Low
Non-fatal stroke	Semaglutide vs placebo (0.87, 0.66–1.16)	4	No concerns	No concerns	No concerns	Some concerns (CI crosses 1)	Some concerns ($I^2 = 44.81\%$)	Not assessable*	Moderate
Non-fatal stroke	Tirzepatide vs Dulaglutide (0.91, 0.59–1.40)	1	No concerns	No concerns	No concerns	Some concerns (wide CI)	Some concerns ($I^2 = 44.81\%$)	Not assessable*	Low
Non-fatal stroke	Tirzepatide vs Efpeglenatide (0.86, 0.35–2.11)	0	No concerns	No concerns	Major concerns (indirect-only)	Major concerns (very wide CI)	Some concerns ($I^2 = 44.81\%$)	Not assessable*	Very low
Non-fatal stroke	Tirzepatide vs Exenatide (0.81, 0.38–1.74)	0	No concerns	No concerns	Major concerns (indirect-only)	Major concerns (very wide CI)	Some concerns ($I^2 = 44.81\%$)	Not assessable*	Very low
Non-fatal stroke	Tirzepatide vs Liraglutide (0.78, 0.36–1.67)	0	No concerns	No concerns	Major concerns (indirect-only)	Major concerns (very wide CI)	Some concerns ($I^2 = 44.81\%$)	Not assessable*	Very low
Non-fatal stroke	Tirzepatide vs Lixisenatide (0.62, 0.27–1.39)	0	No concerns	No concerns	Major concerns (indirect-only)	Major concerns (very wide CI)	Some concerns ($I^2 = 44.81\%$)	Not assessable*	Very low
Non-fatal stroke	Tirzepatide vs Semaglutide (0.79, 0.40–1.57)	0	No concerns	No concerns	Major concerns (indirect-only)	Major concerns (very wide CI)	Some concerns ($I^2 = 44.81\%$)	Not assessable*	Very low

Supplemental Table S26: Certainty of evidence for agent-level network estimates was assessed using the CINeMA framework across key outcomes, with domain judgments and overall confidence ratings reported for each comparison. * Incoherence was not assessable because there were insufficient data to contrast direct and indirect evidence for the same comparison.
